# *β*-adrenergic signaling broadly contributes to LTP induction

**DOI:** 10.1371/journal.pcbi.1005657

**Published:** 2017-07-24

**Authors:** Joanna Jȩdrzejewska-Szmek, Vincent Luczak, Ted Abel, Kim T Blackwell

**Affiliations:** 1 The Krasnow Institute for Advanced Studies, George Mason University, Fairfax, Virginia, United States of America; 2 Department of Biology, University of Pennsylvania, Philadelphia, Pennsylvania, United States of America; SUNY Downstate MC, UNITED STATES

## Abstract

Long-lasting forms of long-term potentiation (LTP) represent one of the major cellular mechanisms underlying learning and memory. One of the fundamental questions in the field of LTP is why different molecules are critical for long-lasting forms of LTP induced by diverse experimental protocols. Further complexity stems from spatial aspects of signaling networks, such that some molecules function in the dendrite and some are critical in the spine. We investigated whether the diverse experimental evidence can be unified by creating a spatial, mechanistic model of multiple signaling pathways in hippocampal CA1 neurons. Our results show that the combination of activity of several key kinases can predict the occurrence of long-lasting forms of LTP for multiple experimental protocols. Specifically Ca^2+^/calmodulin activated kinase II, protein kinase A and exchange protein activated by cAMP (Epac) together predict the occurrence of LTP in response to strong stimulation (multiple trains of 100 Hz) or weak stimulation augmented by isoproterenol. Furthermore, our analysis suggests that activation of the *β*-adrenergic receptor either via canonical (G_s_-coupled) or non-canonical (G_i_-coupled) pathways underpins most forms of long-lasting LTP. Simulations make the experimentally testable prediction that a complete antagonist of the *β*-adrenergic receptor will likely block long-lasting LTP in response to strong stimulation. Collectively these results suggest that converging molecular mechanisms allow CA1 neurons to flexibly utilize signaling mechanisms best tuned to temporal pattern of synaptic input to achieve long-lasting LTP and memory storage.

## Author summary

Long-term potentiation of the strength of synaptic connections is a mechanism of learning and memory storage. One of the most confusing aspects of hippocampal synaptic potentiation is that numerous experiments have revealed the requirement for a plethora of signaling molecules. Furthermore the degree to which molecules activated by the stress response modify hippocampal synaptic potentiation and memory is still unclear. We used a computational model to demonstrate that this molecular diversity can be explained by considering a combination of several key molecules. We also show that activation of *β*-adrenergic receptors by the stress response appears to be involved in most forms of synaptic potentiation, though in some cases unconventional mechanisms are utilized. This suggests that novel treatments for stress-related disorders may have more success if they target unconventional mechanisms activated by *β*-adrenergic receptors.

## Introduction

Synaptic plasticity is one of the cellular mechanisms underlying learning and memory. In the hippocampus, long-term potentiation (LTP) has been implicated not only in acquisition, consolidation and retrieval of spatial memories, but also contextual fear extinction [1–4]. Several neuromodulatory systems contribute to both synaptic plasticity and fear memory [5], including pathological memory retention such as post-traumatic stress disorder (PTSD). One of the most potent regulatory systems is the noradrenergic system, which is activated by arousal, emotion and stress. Experimental evidence shows that norepinephrine is elevated in the hippocampus in mouse models of PTSD [6, 7]; however, its contribution to long term plasticity is unclear and this lack of knowledge hinders the development of treatments for fear memory disorders.

Numerous experiments investigating long-lasting LTP have revealed the requirement for a plethora of signaling molecules (reviewed in [5, 8]). Experimental protocols that induce long-lasting LTP activate diverse signaling pathways, which may interact competitively or cooperatively. For example, long-lasting LTP evoked by multiple trains of high-frequency electric stimulation requires protein kinase A (PKA) only if the inter-train interval is greater than 60 sec [9, 10]. These networks of signaling pathways may converge on common targets, such as extra cellular regulated kinase (ERK), which is required for most forms of long-lasting LTP [11–14]. Alternatively, some components of those signaling pathways are location specific and function in restricted spatial compartments such as spines or dendritic submembrane. Those observations pose the key question of whether this diversity of mechanisms can be explained by collectively considering the combined molecular network.

Another type of unexplained diversity of mechanisms underlying induction of long-lasting LTP is introduced by neuromodulation. To date, *β*-adrenergic receptor (*β*AR) activation has been considered essential for only a subset of experimental protocols, usually for weak electric stimulation. Conversely, commonly used *β*AR antagonists, such as propranolol, do not affect long-lasting LTP elicited by strong electric stimulation.

The idea that *β*AR activation is not essential for long-lasting forms of LTP was undermined by recent experiments suggesting that conventional *β*AR antagonists do not block all downstream signaling pathways. Though *β*ARs typically are coupled with stimulatory G protein (G_s_), phosphorylated *β*ARs decouple from G_s_ and couple with inhibitory G protein (G_i_). Both G_s_-activated and *β*AR coupled to G_i_-activated signaling pathways converge on a common target, ERK [15–17], which is required for long-lasting LTP. The ability of propranolol to recruit ERK [18], suggests that long-lasting LTP evoked by strong stimulation with or without propranolol might require *β*AR signaling to ERK. This hypothesis is supported by recent experiments showing that a complete *β*AR antagonist blocks long-lasting LTP induced by strong electric stimulation [19]. Therefore, *β*AR activation might play a pivotal role for many forms of long-lasting LTP.

To investigate whether the diverse experimental evidence can be unified by considering activation of multiple signaling cascades and address the role of *β*AR activation in occurrence of long-lasting LTP, we develop a spatial, mechanistic model of signaling pathways underlying induction of long-lasting forms of LTP. We evaluate spatio-temporal dynamics of key kinases that activate molecular pathways reported to play an essential role in long-lasting forms of LTP. We show that the combined elevation of several molecules in the spine and in the dendrite can predict the induction of long-lasting LTP, and our results suggest that activation of the *β*ARs may be essential for all forms of LTP. These findings may help unravel the contribution of the noradrenergic system to learning and memory and help with the development of treatments for fear and anxiety disorders.

## Materials and methods

**Fig 1:**
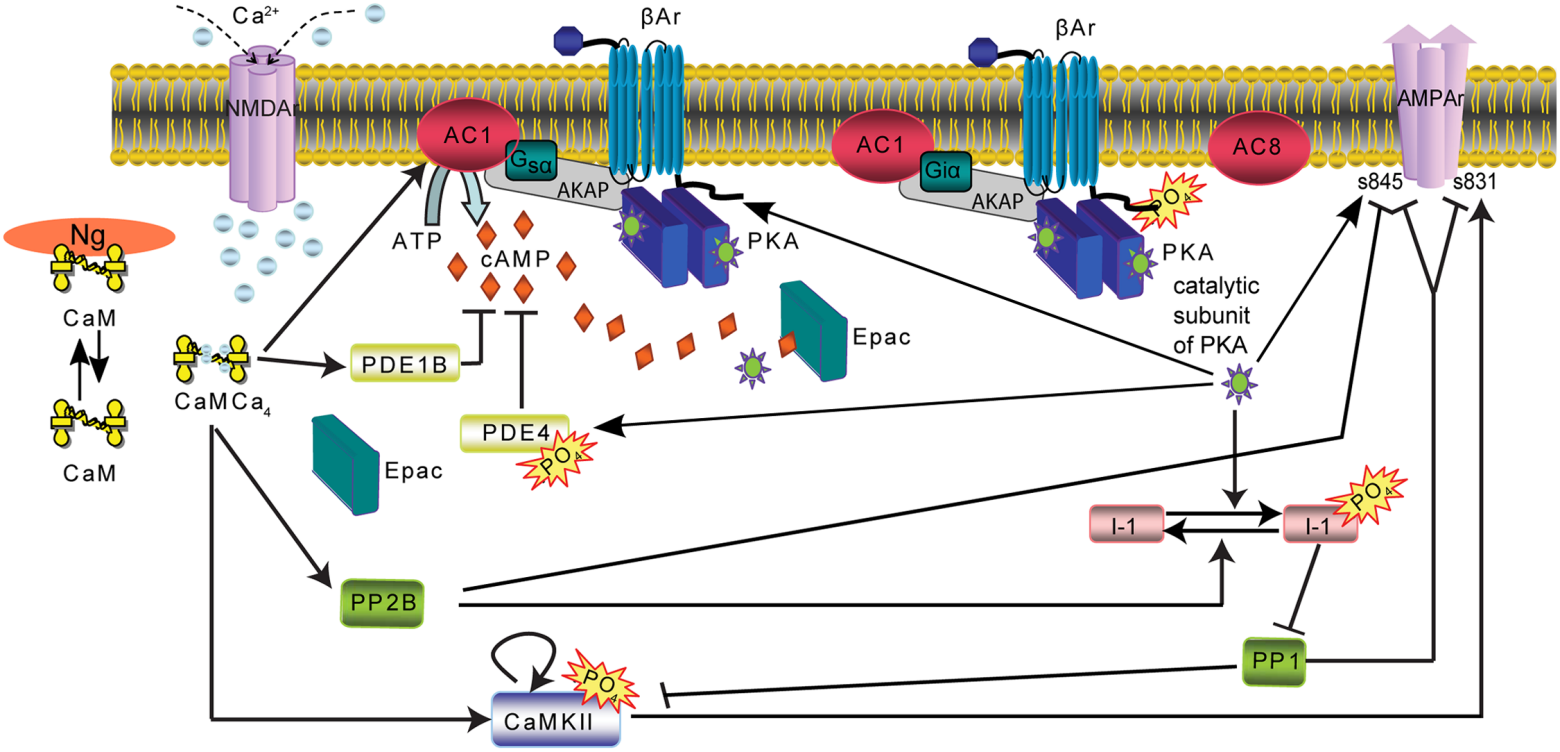
Diagram of postsynaptic signaling pathways. Each arrow is modeled with one or more bimolecular or enzyme reactions. Diffusion is not illustrated in this diagram.

To investigate how temporal pattern of synaptic activation determines which signaling pathways are activated, we employed a multi-compartmental, stochastic reaction-diffusion model of calcium and cAMP activated signaling pathways (Fig 1). The model was adapted from an existing model of a dendrite plus spine of a CA1 hippocampal pyramidal neuron [20]. The signaling pathways included calcium-calmodulin activated molecules, such as calcineurin (PP2B); and phosphodiesterase 1B (PDE1B), cAMP activated molecules: Epac and PKA, and interactions between calcium and cAMP pathways via Inhibitor1. The previously published model [20] was modified by adding neurogranin (Ng) [21], a calmodulin buffer, implicated in LTP and learning [22, 23]. Most importantly we added several pathways downstream of *β*2AR [24] to the model.

*β*ARs in CA1 pyramidal neurons are activated by norepinephrine and mainly couple to stimulatory G proteins [25, 26]. The activated *α* subunit of G_s_ (G_*α*s_ GTP) synergistically enhances cAMP production by calcium-calmodulin bound adenylyl cyclase 1 (AC1). Elevations in cAMP, produced by either prolonged stimulation of *β*2AR or increases in intracellular calcium, activate PKA, which can phosphorylate *β*2AR. There are four sites of heterologous phosphorylation [27] on the *β*2AR [28], whose phosphorylation leads to alternative G protein coupling. In the model, a single phosphorylation event decouples the *β*2AR from G_s_, and the fully phosphorylated *β*2AR then binds inhibitory G protein (G_i_). The *β*2ARs are phosphorylated in a cooperative and distributive manner [29], which yields an ultrasensitive response [30–32]. Note that both G_i_ [15–17] and *β*-arrestin [33] have been implicated in ERK recruitment to the p*β*2AR; thus, in our model the G_i_ binding to p*β*2AR could alternatively represent *β* arrestin binding. Kinetic constants of the model are presented in S1 Table.

**Fig 2:**
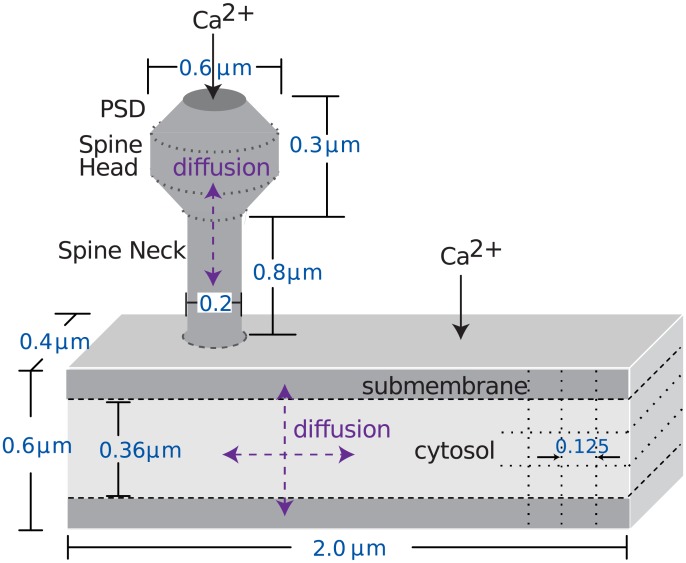
Morphology of dendrite with attached spine and location of calcium influx in the model. Dendritic subvolumes are cuboids, whereas the spine subvolumes are either cylindrical or conical, as portrayed. Dotted lines show part of the subvolumes. Those subvolumes adjacent to the top and bottom surface of the dendrite are considered submembrane subvolumes. Other dendritic subvolumes are part of the cytosol. Calcium injection in a focal dendritic region represents influx through voltage dependent calcium channels. Calcium injection in the PSD represents influx through NMDA receptors and voltage dependent calcium channels in the spine. Diffusion is two-dimensional in the dendrite and one-dimensional in the spine, with reflective boundary conditions at the surface, and diffusion between spine and dendrite.

The morphology of the model comprised one spine attached to a 2 *μ*M dendrite or 8 spines attached to a 20 *μ*M long dendrite with 0.6 *μ*M diameter (Fig 2). In all cases, the dendrite and spines were subdivided into voxels to accurately simulate spatial aspects of signaling molecules. Molecules diffused between spine and dendrite with a coupling coefficient proportional to the surface area of the spine neck. The layer of voxels immediately adjacent to the membrane was considered the submembrane domain. AC (type 1 and 8), PKA holoenzyme, G proteins and the *β*2ARs were localized and anchored both in this submembrane domain and the spine head. The diffusible molecules included cAMP, ATP, calcium, all forms of calmodulin (CaM), CaMKII, *β*2AR agonists and antagonists, Inhibitor-1 and Epac. Their diffusion constants are listed in S2 Table. Initial conditions were either taken from the prior model [20], experimental publications (e.g. quantity of neurogranin [21]), or adjusted to reproduce experimentally measured concentrations of dependent molecules, e.g. the balance of AC and PDE was adjusted to produce a 30 nM basal cAMP concentration [34, 35].

### Stimulation protocols

Different forms of LTP are evoked by different stimulation patterns [36]; thus, we performed simulations using seven, well characterized, stimulation protocols (Table 1). Four of them experimentally elicit L-LTP, one results in an early form of LTP (E-LTP) and the remaining two stimulation protocols do not produce LTP, though one (LFS) elicits brief depression. Electric stimulation of Schaeffer collaterals results in activation of post-synaptic NMDA receptors and action potentials, thus each stimulation pulse was simulated in the model as calcium injection both into the spine to represent NMDA receptors, and into the dendrite to represent activation of voltage dependent calcium channels. Electric stimulation in the hippocampus is accompanied by norepinephrine release [37], which was modeled as ligand influx. Bath applied isoproterenol (ISO) was simulated by injecting sufficient ISO to produce a 1 *μ*M concentration. We started stimulation after 300 sec of simulation to ensure the model had reached equilibrium. Steady state was confirmed by running simulations for 900 sec in the absence of stimulation and visually assessing that activity of each molecular specie was stationary.

**Table 1:** Experimental protocols and their characteristics. n.a. stands for not applicable. Protocol provides the abbreviation that is used throughout the article. Description gives brief explanation of experimental protocol. Outcome indicates experimentally observed outcome of the protocol: early phase of LTP (E-LTP), a long-lasting form of LTP or no change. Molecular dependence lists which molecules were experimentally shown essential.

Protocol	Description	Outcome	Molecular dependence
LFS	180 sec of 5 Hz	brief depression [12, 55]	n.a.
ISO	bath applied 1 *μ*M of isoprotenerol	no change [12, 55]	n.a.
HFS	1 sec of 100 Hz (1 train)	E-LTP [56]	CaMKII [57]
4xHFS-3s	4 trains of HFS with 3 sec inter-train interval	E-LTP, long-lasting LTP	CaMKII [58]
ISO+HFS	bath applied 1 *μ*M of isoprotenerol 10 minutes before HFS	E-LTP, long-lasting LTP [14]	Epac, ERK [14]
4xHFS-80s	4 trains of HFS with 80 sec inter-train interval	E-LTP, long-lasting LTP	PKA [9, 59], ERK [11]
ISO+LFS	bath applied 1 *μ*M of isoprotenerol 10 minutes before LFS	long-lasting LTP [12, 55]	PKA, ERK [12]

Several data sources were used to adjust calcium pulse amplitudes for all stimulation protocols. To stimulate calcium influx during 100 Hz trains of electric stimulation (HFS), we used release probabilities from [38] which provides changes in the amplitudes of calcium pulses in the spine during high frequency trains. We assumed that amplitudes of consecutive calcium pulses in the dendrites are uniform, because they result from full height action potentials. To calculate absolute amplitudes of calcium pulses, we constrained calcium concentration in the spine and in the dendrite to match experimental data [39]: 10 *μ*M in the spine and 2 *μ*M in the dendrite. This pattern of calcium pulses was used in all stimulation protocols using trains of HFS: 1 train of 100 Hz (HFS), four trains of 100 Hz given 3 sec apart (4xHFS-3s), four trains of 100 Hz given 80 sec apart (4xHFS-80s) and bath applied ISO followed by 1 train of 100 Hz (ISO+HFS). For the 5 Hz (LFS) stimulation protocol, spine calcium pulses were of the same amplitude, and equal to the amplitude of the first pulse of the HFS train [39].

In order to estimate the temporal pattern and amplitude of neuromodulation elicited by electric stimulation, we used a model (Eq 1) describing vesicle release [40]. This model assumes that synaptic resources can be found in three states: inactive (*I*), recovered (*R*) and effective (*E*; released). *u* represents release probability, which decays with a time constant *τ*_*f*_ and increases with each action potential (AP) by a fraction of *U*_SE_. After the arrival of the AP a fraction of recovered resources (*uR*) becomes effective (*E*) i.e. gets released. Effective resources, *E*, become inactive with a time constant *τ*_*i*_. Inactive resources, *I*, recover with a time constant *τ*_*r*_. The Dirac delta function is denoted as *δ*(*t* − *t*_AP_) and has value 1 at *t* = *t*_AP_ and 0 otherwise. *A*_*SE*_ is the absolute synaptic efficacy (response amplitude produced by complete release of all the neurotransmitter). We tuned the vesicle release model on experimental data to voltammetric measurements of norepinephrine release in the rat Ventral Bed Nucleus Stria Terminalis following electric stimulation of noradrenergic projection pathways [41] (S6 Table). Using this model we estimated norepinephrine release for stimulation patterns in Table 1. The spatial distribution of norepinephrine during and following release was in agreement with a spatial gradient of neuromodulators [42], namely a high concentration in the spine release site (1 *μ*M) and lower at the dendrite.

$$\begin{array}{c} \begin{array}{cc} & \frac{dR}{dt}=\frac{1-R-E}{\tau_{r}\;}-uR\delta \left(t-t_{\text{AP}}\;\right)\\ & \frac{dE}{dt}=-\frac{E}{\tau_{i}\;}-uR\delta \left(t-t_{\text{AP}}\;\right)\\ & \frac{du}{dt}=-\frac{u}{\tau_{f}\;}+U_{\text{SE}}\;*\left(1-u\right)\delta \left(t-t_{\text{AP}}\;\right)\\ & \text{Amplitude}\;\text{of}\;\text{neurotransmitter}\;\text{release}\;\text{in}\;\text{response}\;\text{to}\;\text{single}\;\text{AP}=A_{\text{SE}}\;E \end{array} \end{array}$$ (1)

PKA phosphorylates NMDA receptors, which increases the amplitude of calcium influx through these receptors [43, 44]. This enhancement of NMDA mediated calcium influx has been observed with bath application of ISO. Thus, for the case of ISO+LFS, calcium influx was increased by 50% [45].

We modeled propranolol (1 *μ*M; [46]) ICI-118,551 (100 nM; [19]) and carvedilol (10 *μ*M [47]) by allowing it to bind the *β*2AR [48] (S1 Table), and then both propranolol- and carvedilol-bound *β*2AR were able to bind with G_i_ and form a target representing ERK activation. Binding affinity was constrained so that carvedilol produces one third the G_i_ bound *β*2AR compared to that of isoproterenol as has been measured experimentally [18].

### Simulation

We used a stochastic simulation technique, as many molecular populations are small. In such case activations fluctuate greatly about the mean within such small compartments [49, 50]. Similarly, diffusion of second messenger molecules out of the spines and along the thin dendrites is subject to random variation. The model was implemented using an efficient mesoscopic stochastic reaction-diffusion simulator NeuroRD [51], version 2.1.10, because the large numbers of molecules in the morphology described (Fig 2) made tracking individual molecules in microscopic stochastic simulators computationally expensive. This simulator uses reflective boundary conditions (molecules attempting to diffuse out of the morphology were reflected back into the morphology). Model simulations used a time step of 2.9 *μ*s. A single simulation of 900 sec (of the dendrite with 1 spine) takes 4.5 days on a Intel Xeon CPU E5-2620 2.00GHz processor.

Based on results from our prior studies, simulations were repeated four or eight times using a different random seed. Eight simulations were used for stimulation protocols whose signature exhibited a large standard deviation relative to the mean. To determine whether the combination of stimulation and *β*AR ligand would induce L-LTP or not, we analyzed the duration of combined molecular activations (signatures) in the spine and in the dendrite above their respective thresholds. The statistical analysis used SAS (version 9.4, SAS Institute, NC). Student’s T test (SAS procedure TTEST) was applied to each condition to evaluate whether the duration above threshold was significantly greater than the duration threshold of 10 sec. For the multi-spine simulations, we used the SAS procedure GLM to perform a two-way analysis of variance using condition (adjacent or separate) and stimulation (spine was stimulated or not) as factors. All model simulation files are available from modelDB (https://senselab.med.yale.edu/ModelDB/showModel.cshtml?model=190304).

### Model validation

**Fig 3:**
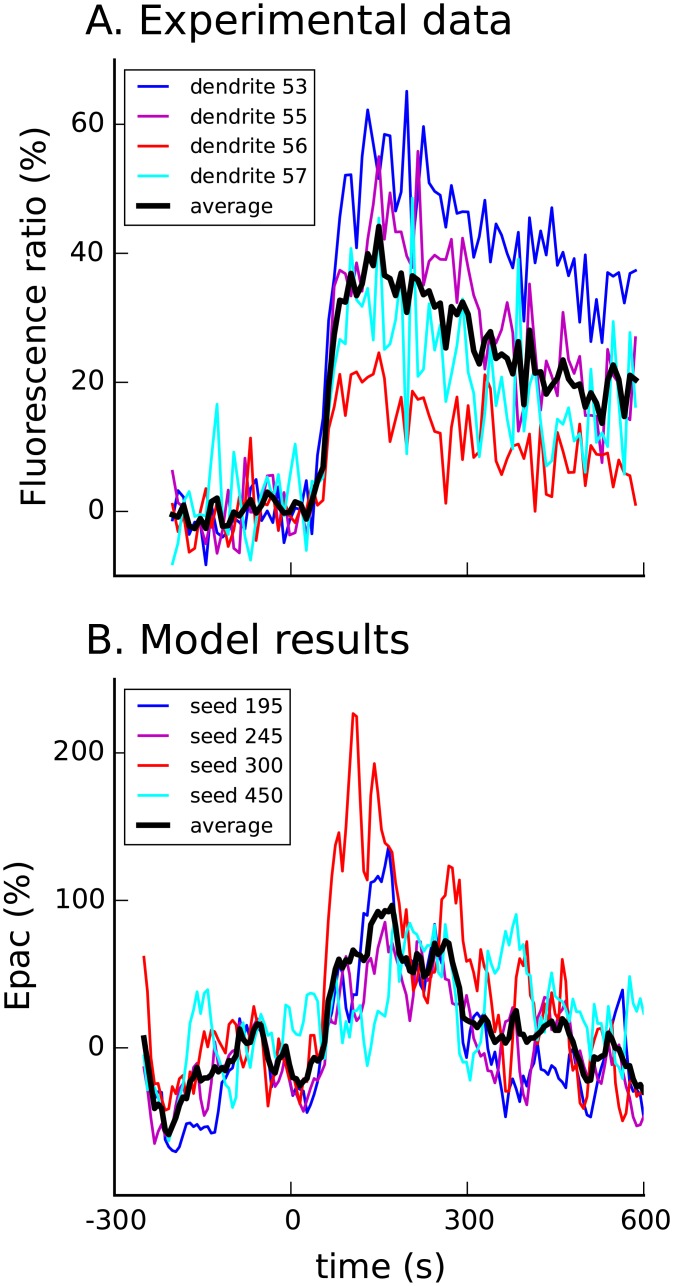
Decay of Epac activity and maximum Epac activity in response to 1 *μ*M ISO bath in the model over-expressing Epac (B) is similar to dynamics of experimentally measured epac-sh150 fluorescence (A) [52]. Different traces show different experiments (A) or different random seeds (B).

We validated the model by comparing activity of AMPA receptor (AMPAR) phosphorylation and PKA-mediated G_s_-G_i_ switching with independent, published experimental results. To validate the PKA-mediated G_s_-G_i_ switching we simulated bath application of 1 *μ*M of isoproterenol in a model over-expressing Epac (8 time the amount used in other simulations). The model’s Epac activity was compared with the response of genetically encoded Epac-sh150 (monitoring cAMP activity in hippocampal CA1 neurons) to 1 uM ISO [52]. Fig 3 shows the model’s Epac activity and fluorescence traces of distal dendrites in response to bath application of 1 *μ*M of isoproterenol (ISO) and confirms that the model accurately captures the decay in cAMP activity while isoproterenol is still present due to phosphorylation of *β*ARs (and phosphodiesterases). For comparison we chose fluorescence traces of small, tertiary dendrites, which had similar diameter to the dendrite diameter used in the model.

Next we validated the model of AMPAR phosphorylation by comparing phosphorylation AMPAR at Serine 845 and Serine 831 to experimentally measured values. In the model bath application of 1 *μ*M of ISO yields 200% increase in phosphorylation of Serine 845 and no discernible phosphorylation of Serine 831, which is in agreement with values reported in hippocampal CA1 neurons after bath applying 1 *μ*M of ISO [53, 54]. These comparisons confirm the parameters describing inactivation mechanisms (both G_s_-G_i_ switching and PDE4 phosphorylation) of cAMP and PKA activity for AMPAR.

## Results

Our goal was to explain the diverse literature on molecular dependence of long-lasting forms of LTP induction. We evaluated whether the spatio-temporal dynamics of molecular signaling pathways can explain and predict which stimulation patterns produce long-lasting LTP. We constructed a model of signaling pathways (Fig 1) that regulate long-lasting forms of LTP in hippocampal CA1 pyramidal neurons in NeuroRD [51] using the morphology of a dendrite with one spine (Fig 2). We simulated seven experimental protocols (Table 1), four of which elicit long-lasting forms of LTP, one of which results in E-LTP, and two of which cause no lasting change in synaptic efficacy. Our goal was to create a simple set of equations to explain all the outcomes, and also molecular dependence of seven protocols. In designing the equation, we concentrated on the activity of molecular species that are implicated in spine-specific and dendrite-specific changes and accompany long term plasticity.

### Spine and dendritic molecular signatures required to predict plasticity

We quantified the spatio-temporal dynamics of molecular species that are known to play a role in the induction of long-lasting forms of LTP, including PKA [9, 12, 55, 60], calcium-calmodulin-dependent protein kinase II (CaMKII) [58, 61–63] and exchange protein directly activated by cAMP (Epac) [14]. These molecules were activated either by calcium pathways or by the *β*AR coupling either to G_s_ or G_i_. We empirically determined two equations that we called ‘signatures’ to predict the occurrence of long-lasting LTP. The first one summed normalized activity of key molecular species in the spine, the second one summed normalized activity of key molecular species in the dendrite. We assumed that if the experimental protocol enhanced activity of key molecular species in the spine, then spine specific changes would be induced and, similarly, if the experimental protocol enhanced activity of key molecular species in the dendrite, then dendrite specific changes would be induced. To evoke long-lasting forms of LTP both spine specific and dendrite specific changes needed to be induced.

The spine molecular signature trace (referred to as the spine signature) evaluates the initiation of plasticity processes in the spine by calculating time dependent increases in CaMKII, Epac, and PKA activity in the spine:

$$\begin{array}{c} S_{\text{spine}}\left(t\right)=\frac{\Delta \text{pCaMKII}(t)}{\max\Delta \text{pCaMKII}}+\frac{\Delta \text{Epac}(t)}{\max\Delta \text{Epac}}+\frac{\Delta \text{PKA}(t)}{\max\Delta \text{PKA}} \end{array}$$ (2)

where ΔEpac(*t*) is the fold increase in cAMP bound Epac, ΔpCaMKII(*t*) is fold increase in phosphorylated CaMKII,

ΔPKA(*t*) is the fold increase in phosphorylation of PKA targets. Max Δ*X* is a normalization value equal to the maximum activation of molecular specie *X* among the seven control protocols, where the maximum activation was calculated as the mean (over trials) of the peaks (for each trial). If the spine signature exceeds its threshold for more than 10 sec, spine-specific changes are induced.

The dendritic signature represents spatially non-specific plasticity processes, and takes into account molecular species: PKA, Epac and CaMKII:

$$\begin{array}{c} \begin{array}{cc} S_{\text{dendrite}}\left(t\right) & =\frac{\Delta \text{Epac}(t)}{\max\Delta \text{Epac}}+\frac{\Delta \text{pCaMKII}(t)}{\max\Delta \text{pCaMKII}}\\ & +\frac{\Delta (\text{pInhibitor}1(t)+\text{pPDE}4(t))}{\max\Delta (\text{pInhibitor}1+\text{pPDE}4)}+\frac{\Delta G_{i}\left(t\right)}{\max\Delta G_{i}}, \end{array} \end{array}$$ (3)

For the dendritic signature, the PKA activity is subdivided into two terms: inhibitory G protein (G_*i*_(*t*)) which represents phosphorylated *β*2AR, and other phosphorylated PKA targets: Inhibitor-1 and PDE4. We have subdivided the PKA activity into these two parts to evaluate the role of G_s_-G_i_ switching (and *β*-arrestin) in synaptic plasticity, and also to evaluate the role of novel *β*2AR antagonists. Δ(pInhibitor1(*t*) + pPDE4) represents PKA phosphorylation of other phosphoproteins included in the model for LTP induction. If the dendritic signature exceeds its amplitude threshold for more than the 10 sec duration threshold, dendrite-specific changes are induced.

We chose a relatively short duration threshold as it has been shown that the temporal window of CaMKII activation required for synaptic plasticity and learning is narrow [64], less than 1 minute. To induce long-lasting forms of LTP, both the spine- and dendrite-specific changes must be induced.

#### Molecular signatures explain both electrically and chemically induced LTP

**Fig 4:**
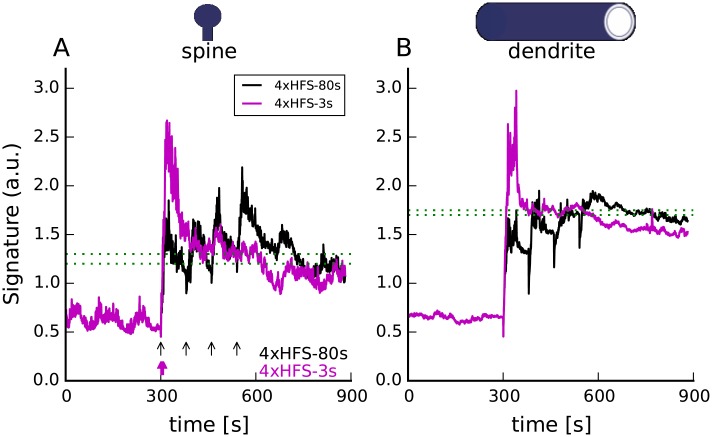
The molecular signatures correctly predict that both 4xHFS-80s and 4xHFS-3s will elicit a long-lasting form of LTP. Both spine (A) and dendritic (B) signatures of both protocols cross their respective thresholds. Black and magenta arrow indicate the time at which a 1 sec train of 100 Hz stimulation is given to the model. For all panels dashed green lines represent the range of threshold that correctly predicts the plasticity outcome. Traces show results of representative simulations.

**Fig 5:**
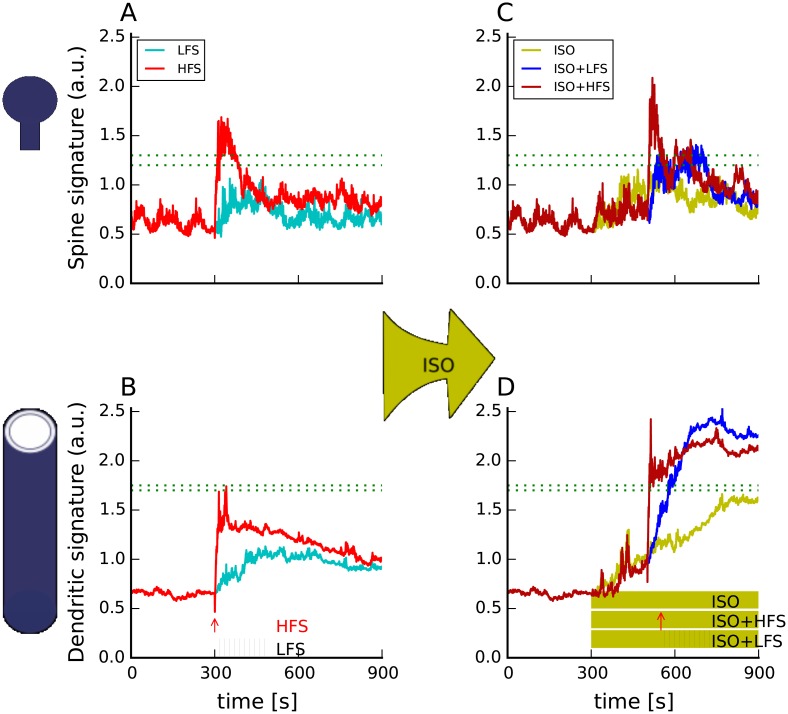
The molecular signatures correctly predict *β*AR activation will transform weak stimulation (LFS, HFS, signatures in panels A,B) into a long-lasting form of LTP (signatures in panels C,D). The effect of the *β*AR activation is visible mainly in the dendrite (B, D), where it elevates the dendritic signature above the threshold for both ISO+LFS and ISO+HFS. It also provides necessary cAMP elevation to allow the spine signature (A, C) of ISO+LFS to cross the threshold. In (B,D) vertical black lines represent the approximate time of the 3 min train of 5 Hz stimulation. Red arrow shows the timing of the 100 Hz train of stimulation. In D, the mustard rectangle shows the time and duration of 1 uM ISO application. For all panels dashed green lines represent the range of threshold that correctly predicts the plasticity outcome. Traces show results of representative simulations.

The first question addressed was whether a single set of empirically derived thresholds could predict the outcome of seven different experimental protocols (control protocols) without a change in the models parameters. Figs 4 and 5 show that indeed there is a range of thresholds for both the spine and dendritic signature, which allows for predicting long-lasting forms of LTP. Furthermore, simulations of HFS (Fig 5) demonstrate that separate spine and dendritic signatures are needed. Specifically, the signatures for HFS, which does not produce a long-lasting form of LTP, exceed the spine threshold but not the dendritic threshold. In summary our model with spine and dendritic signatures correctly predicts the plasticity induction for control protocols, whereas a single signature would have given wrong predictions. This demonstrates that the spatial aspect of the model is crucial.

Another question we investigated was how bath application of ISO, which activates the *β*ARs, transforms weak electric stimulation, such as LFS or HFS, into a protocol that evokes long-lasting forms of LTP. Fig 5 reveals that indeed ISO transforms HFS and LFS into long-lasting LTP. Though the spine signature for HFS crosses the threshold (Fig 5A) ISO is needed for the dendritic signature to cross the threshold (Fig 5B and 5D). These signatures suggest that HFS alone can induce the spine-specific changes required for plasticity, which is consistent with experiments showing that HFS can “tag” the synapse [65, 66], but that ISO is required to activate processes in the dendrite or soma required for long-lasting forms of LTP. In contrast to HFS, ISO enhances both the spine and dendritic signature for LFS, suggesting that LFS alone is insufficient to induce either spine-specific or dendrite-specific changes. These simulations make the experimentally testable prediction that HFS is sufficient for synaptic tagging, but that LFS is not.

#### Signature validation

To validate the signatures and also evaluate the PKA dependence and temporal sensitivity of long-lasting forms of LTP, we performed an additional set of simulations in the presence of specific PKA inhibitors. Bath application of PKA inhibitors was simulated by eliminating activity of the PKA catalytic subunit. For all simulated protocols, we calculated both spine and dendritic molecular signatures and used the same thresholds determined for the previous set of simulations.

**Fig 6:**
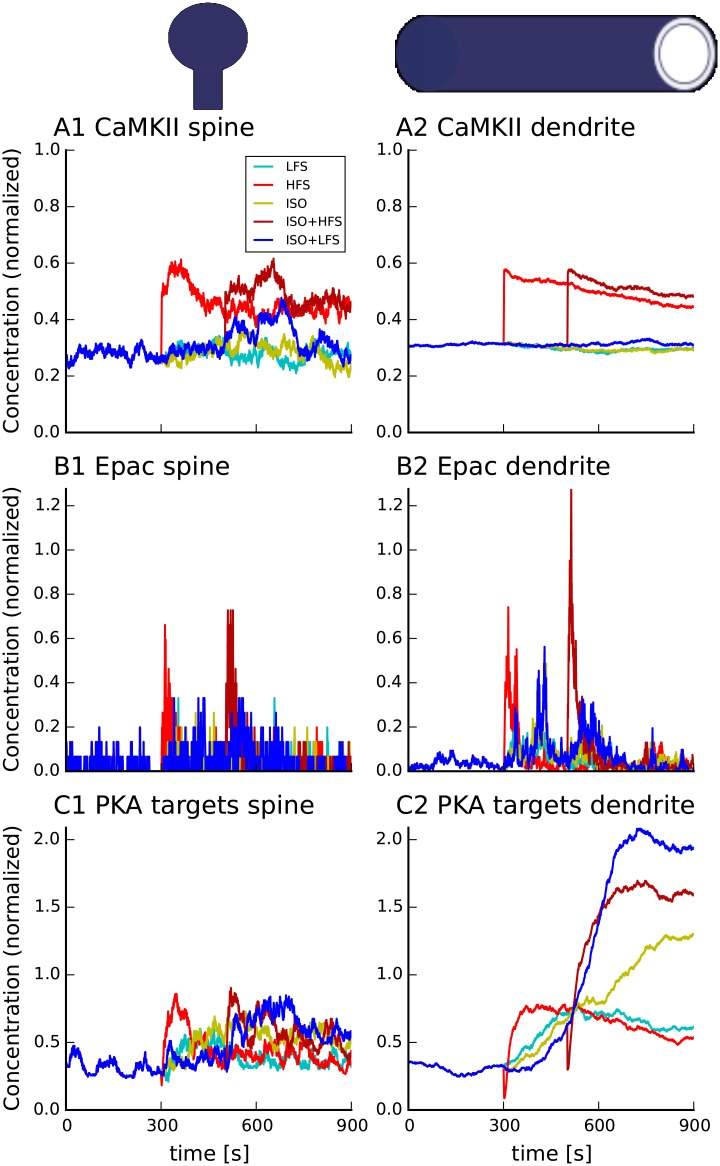
Molecular signatures in the spine (C) and dendrite (D) predict that inhibiting PKA blocks long-lasting forms of LTP for PKA-dependent stimulation protocols. As seen in panels B1 and B2, in the presence of PKA inhibitors, an elevation in Epac activity compensates for PKA for 4xHFS-3s and ISO+HFS. PKA inhibition slightly lowers CaMKII in the spine (A1) and dendrite (A2). Legend in panel B2 applies to all panels. Onset of electrical stimulation is 300 sec for all protocols. ISO application begins at 300 sec for ISO+HFS and ISO+LFS. Trains of 100 Hz stimulation are separated by 80 s for the 4xHFS-80s and 3s for the 4xHFS-3s protocols. The dashed green lines represent the range of threshold, which correctly predicts the plasticity outcome. Traces show results of representative simulations.

Consistent with experiments [9, 14], blocking PKA lowers either the spine (Fig 6C) or dendritic (Fig 6D) signature below threshold for all PKA-dependent forms of plasticity. Blocking PKA activity lowers the spine signature for ISO+LFS, but not that of the other protocols. Blocking PKA lowers the dendritic signature for 4xHFS-80s, so that it no longer crosses the threshold. Collectively, the model correctly predicts that blocking PKA will block long-lasting LTP induced by both 4xHFS-80s, and ISO+LFS, but will not block long-lasting LTP produced by 4xHFS-3s or ISO+HFS. In addition, these simulations demonstrate that molecular processes in different spatial compartments can diverge and make different contributions to the induction of L-LTP.

#### Molecular signatures explain PKA dependence

**Fig 7:**
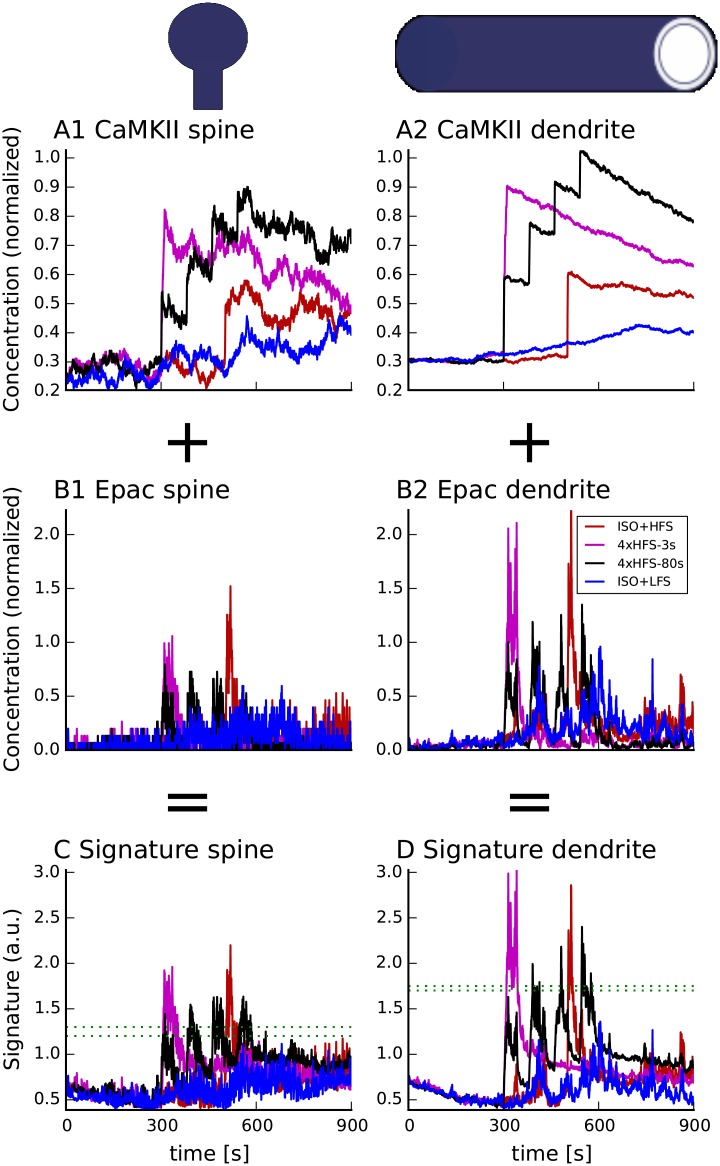
Traces of molecular components of the signature reveal that LFS, but not HFS, requires PKA for the signature to exceed the threshold for induction of long-lasting LTP. PKA is not required for long-lasting LTP induced with ISO+HFS because ISO increases Epac strongly for HFS. Legend in panel A1 applies to all panels. (A) CaMKII activity in the spine (A1) and dendrite (A2),(B) Epac activity in the spine (B1) and dendrite (B2), (C) PKA activity in the spine (C1) and dendrite (C2). Panels C1 and C2 show activity of all PKA targets, including G_*iβγ*_, which represents *β*2AR phosphorylation. Note that amplitude of PKA target at 900 sec in panel C2 depicts G_*iβγ*_ levels. At 900 s both pPDE4 and pInhibitor1 activity has returned back to basal. Stimulation for the 5 protocols are illustrated in Fig 5. Traces show results of representative simulations.

We evaluated molecular dependence of LTP by examining distinct molecular components of the spine and dendrite plasticity signature in the control protocols and in the PKA blocked protocols. Prior research revealed that ISO+HFS requires Epac, but not PKA [14], whereas ISO+LFS requires PKA [12]. Consistent with these experimental observations, the model shows that both ISO+LFS and ISO+HFS need cAMP activated molecules provided by ISO: either PKA (Fig 7C) or Epac (Fig 7B), because CaMKII activity is too small (Fig 7A). For ISO+LFS (but not ISO+HFS), PKA is specifically needed to exceed the threshold, because Epac is too small.

ISO enhances both the spine and dendritic signatures for LFS by enhancing CamKII activation (Fig 7A and 7B), which has also been observed experimentally [67]. The enhancement of CaMKII is caused by both higher calcium influx due to NMDAR phosphorylation by PKA and subsequent inhibition of PP1. For HFS, however, PP2B activation is so strong that PP2B immediately dephosphorylates all Inhibitor-1, regardless of whether phosphorylated Inhibitor-1 has been increased by ISO application. In other words, the dephosphorylation of Inhibitor-1 is stronger for ISO+HFS than HFS alone. Consequently CaMKII activation is the same for both HFS and ISO+HFS.

Examination of molecular components of the signatures for the blocked PKA protocols helps to further understand the role of PKA and Epac in long-lasting forms of LTP. Blocking PKA reduces PDE4 activity [68], which increases cAMP and Epac activity. The increase in Epac is sufficient to compensate for lack of PKA for both ISO+HFS and 4xHFS-3s (Fig 6B). Epac does not compensate for PKA for the ISO+LFS case, because the low calcium influx with LFS does not activate sufficient CaMKII (Fig 6A) compared to ISO+HFS. Blocking PKA slightly lowers CaMKII in the spine (Fig 6A), but not considerably as would be expected [69], possibly because low Inhibitor-1 levels in the CA1 region of the hippocampus.

#### *β*2AR is a critical PKA target for induction of long-lasting forms of LTP

PKA phosphorylation of *β*2AR has been suggested to be critical for hippocampus-dependent learning and long-lasting forms of LTP [19]. PKA-mediated G_s_-G_i_ switching is potentially relevant for all long-lasting forms of LTP because electric stimulation is accompanied by a release of norepinephrine from locus coeruleus neuron terminals [37]. Though propranolol does not block long-lasting LTP induced by 4xHFS-80s [46], this does not rule out *β*2AR involvement as propranolol is an incomplete antagonist that allows some ERK recruitment [18].

This experimental evidence raises the critical question of whether *β*AR activation is required for all long-lasting forms of LTP. To answer this question we simulated the response to novel *β*AR antagonists in combination with electrical stimulation. We simulated 4xHFS-80s with bath applied propranolol (does not stimulate cAMP production but allows 10% ERK recruitment compared to bath applied ISO [18]) or ICI-118,551 (a complete antagonist [18]). We also simulated LFS and single and multiple trains of HFS preceded by bath application of carvedilol, which does not stimulate cAMP production, but does allows 30% ERK recruitment compared to ISO [18].

**Fig 8:**
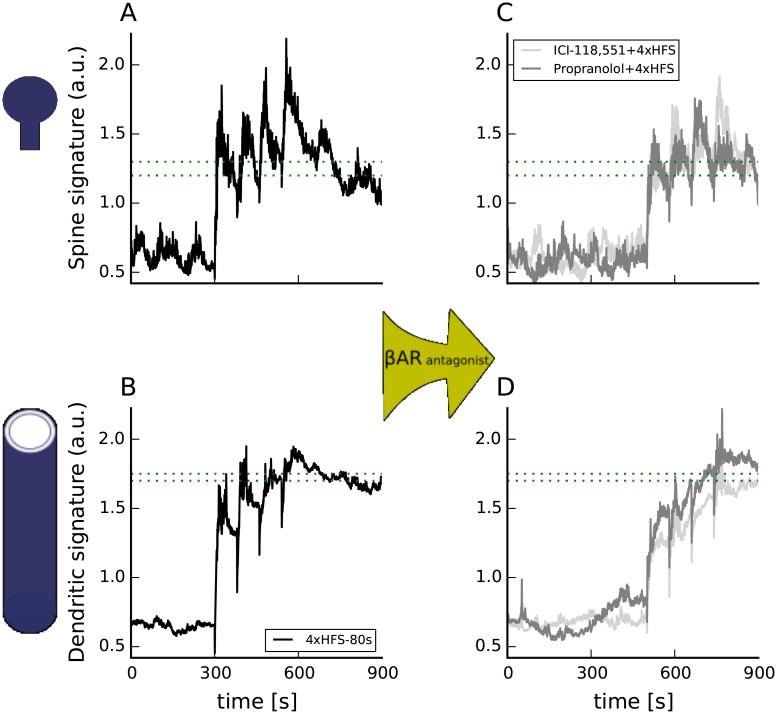
*β*AR activation is critical in the dendrite, but not in the spine to induce L-LTP using 4 trains of 100 Hz spaced 80s apart. Inhibition of *β*AR activation by ICI-118,551, a *β*2AR antagonist, blocks a PKA-dependent long-lasting form of LTP (4xHFS-80s) as the dendritic signature only briefly exceeds the threshold. Propranolol (C, D, gray trace), which recruits some ERK [18], does not abolish long-lasting LTP [46] as both signatures exceed the threshold for more than 10 sec. (A,B) show spine and dendritic signatures of 4xHFS-80s in control conditions. (C,D) show effect of ICI-118,551 or propranolol. Electrical stimulation begins at 300 sec in (A,B) and 500 sec in (C,D); propranolol or ICI-118,551 is applied beginning at 300 sec in (C,D). The dashed green lines represent the range of threshold which correctly predict the plasticity outcome. Traces show results of representative simulations.

Fig 8 shows that *β*AR is necessary for induction of long-lasting LTP even though propranolol does not block L-LTP induction. *β*AR stimulation is not required to elevate cAMP in the spine, as the signature crossed the threshold in the presence of ICI-118,551, because calcium elevation in the spine is sufficient to produce enough cAMP (Fig 8A and 8C). Simulations suggest that ICI-118,551, but not propranolol reduces the dendritic signature to below the upper threshold, indicating the importance of non-canonical pathways activated by the *β*AR for induction of long-lasting forms of LTP. The difference in dendritic signature between propranolol and ICI-118,551 is small because the difference in these two antagonists with respect to ERK recruitment is rather small [18]. The difference in signatures would be larger if ERK recruitment by propranolol were larger in brain slices compared to HEK-293 cells. These simulations make the experimentally testable prediction that ICI-118,551 will likely block long-lasting LTP induced by 4xHFS-80s. These simulations also predict that ICI-118,551 will not block synaptic tagging (because the spine signature exceeds the threshold after the 2nd train of HFS), and further demonstrate the difference between spatial compartments.

**Fig 9:**
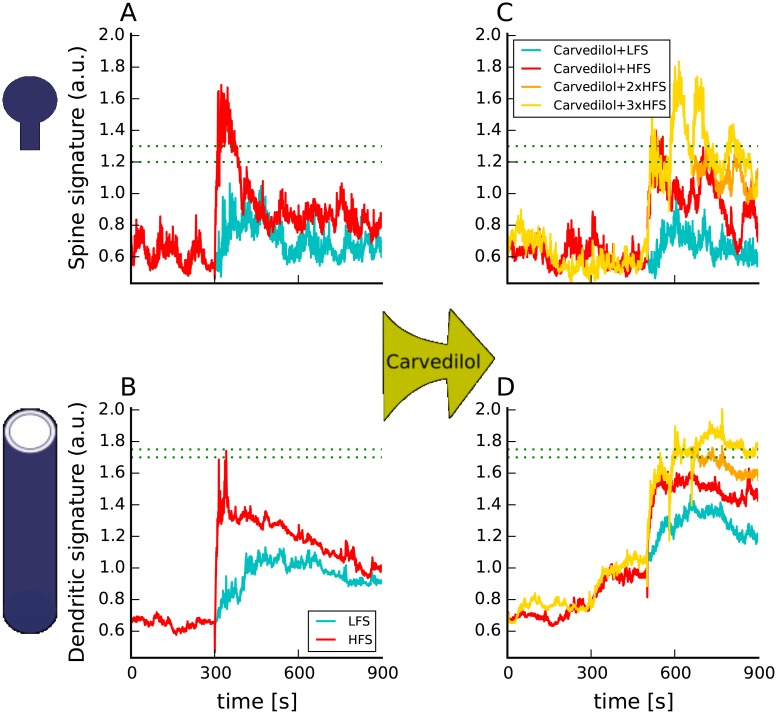
Molecular signatures of experimental protocols eliciting long-lasting forms of LTP using novel *β*2AR antagonist demonstrate the role of non-canonical *β*AR-activated pathways in induction of long-lasting LTP. Spine signature (A,C) predicts that carvedilol will not support a long-lasting form of LTP elicited using HFS and LFS. For HFS, calcium influx due to one 100 Hz train of electric stimulation might not be sufficient to elevate the spine signature above threshold, but two trains are sufficient (C). Dendritic (B,D) signatures of weak electric stimulation are elevated after bath applying carvedilol, but do not cross the threshold. Adding a second train of HFS further elevates dendritic signature but not above the threshold. Only the addition of the third train elevated dendritic signature above its threshold, suggesting that Carvedilol+3xHFS-80s will elicit L-LTP. Trains of HFS begin at 300 sec and repeat at 380s for 2xHFS, and 380s and 460s for 3xHFS in (A,B); trains of HFS begin 250s later in panels (C,D) with carvedilol applied at 300 sec. The dashed green lines represent the range of threshold which correctly predict the plasticity outcome.

To further investigate the role of canonical and non-canonical *β*AR signaling in L-LTP induction we simulated bath application of carvedilol followed by weak stimulation: either HFS or LFS (Fig 9). Carvedilol does not stimulate G_s_, but does allow roughly three times more ERK than propranolol [18]. Spine and dendritic molecular signatures show that carvedilol can not substitute for ISO when paired with either HFS or LFS, and that G_s_ signaling is required to induce L-LTP with weak stimulation. The spine signatures of both the Carvedilol+HFS and Carvedilol+LFS are lower than for weak stimulation alone, because carvedilol blocks binding of norepinephrine, which is released with weak stimulation alone. Furthermore, stimulation protocols that yield low calcium concentration, such as LFS, need additional G_s_ stimulation from canonical *β*AR-activated pathways for the spine signature (Fig 9A) to cross the threshold. In the dendrite, G_i_ recruited by carvedilol binding to *β*AR is too low to compensate for the absence of G_s_ and thus the dendritic signatures for both carvedilol+HFS and carvedilol+LFS do not cross the threshold (Fig 8D). To see how much additional calcium influx is necessary to compensate for *β*AR activation in the dendrite we simulated bath application of carvedilol followed by 2 and 3 trains of HFS with 80 sec inter-train interval (Carvedilol+2xHFS and Carvedilol+3xHFS). Adding one more train of HFS increased the spine signature, resulting in the spine signature definitely crossing its threshold. Two trains did not, however, provide enough calcium for the dendritic signature to cross its threshold. Adding the third HFS train elevated dendritic signature above its threshold, showing that high enough calcium can substitute for G_s_ stimulation. Thus Carvedilol+3xHFS is equivalent to Propranolol+4xHFS. In summary, the model makes the experimentally testable prediction that carvedilol will not support long-lasting LTP induced by either LFS, HFS or 2xHFS; it will, however support L-LTP for 3xHFS. Collectively, these model results, if supported by experimental tests of the model predictions, suggest that *β*AR stimulation is required for all forms of L-LTP, though in some cases G_i_ recruitment instead of canonical G_s_ activation is required.

### E-LTP

**Fig 10:**
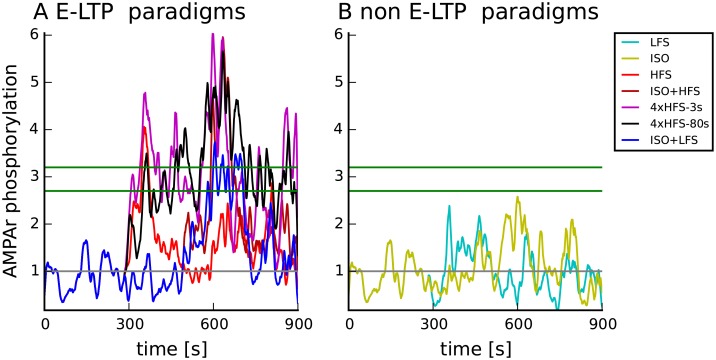
Changes in AMPA receptor phosphorylation (pAMPAR) caused by stimulation protocols from Table 1 correlate with induction of E-LTP. Both panels show phosphorylation at Serine 845, Serine 831 or both, relative to the steady state phosphorylation. (A) Stimulation protocols that elicit E-LTP experimentally. HFS, ISO+HFS, 4xHFS-3s, 4xHFS-80s and ISO+LFS elicit E-LTP. (B) Stimulation protocols that do not elicit E-LTP experimentally. Green lines depict range of thresholds producing correct prediction of E-LTP. Grey horizontal line depicts no change. Traces show results of representative simulations.

The spatial approach allowed us to monitor changes in the phosphorylation of the AMPA receptor subunit GluA1 (AMPAR) in the PSD (Fig 2). We monitored AMPAR phosphorylation (pAMPAR) because it is correlated with E-LTP [70]. To evaluate induction of E-LTP for the seven control protocols, the only additional parameter added was a threshold on AMPA receptor phosphorylation. HFS, ISO+HFS, 4xHFS-3s, 4xHFS-80s and ISO+LFS each cause three-fold increases in phosphorylation of AMPA receptors resembling E-LTP (Fig 10A), whereas ISO causes a smaller increase in phosphorylation of AMPA receptors (Fig 10B), which is in agreement with [71]. Thus, though explaining E-LTP was not a goal of the model, an emergent property was that the model correctly predicts the development of E-LTP. Because only a single additional parameter was added to evaluate the outcomes of seven stimulation protocols, these results are considered an additional validation of the model.

### Stimulation of segregated spines helps preserve spatial specificity

A question of major importance for information processing is which events triggered by synaptic plasticity are spatially specific. Recent experiments using glutamate uncaging at single spines suggest that uncaging induced structural plasticity is spine specific [72]. On the other hand, some molecules, such as Ras, can diffuse into nearby spines, reducing the threshold for LTP at those spines [73, 74]. In addition to spatial specificity, other experiments suggest that stimulation of multiple spines may either cooperate with each other [75] or compete for resources [74]. Thus, the next set of simulations investigated whether electrically induced synaptic plasticity exhibits spatial specificity, i.e., what is the extent of diffusion of key molecules to adjacent spines. We used a 20 *μ*M dendrite with 8 dendritic spines, applied 4xHFS-80s and evaluated stimulation of two adjacent spines (1.5 *μ*m apart) and two non-adjacent (8 *μ*m apart), i.e. separated, spines. Because the model is intrinsically a spatial model, extension of the morphology to a larger dendrite with additional spines requires no changes to reaction rates, molecule concentrations and surface densities, or the equation and thresholds for the signatures.

**Fig 11:**
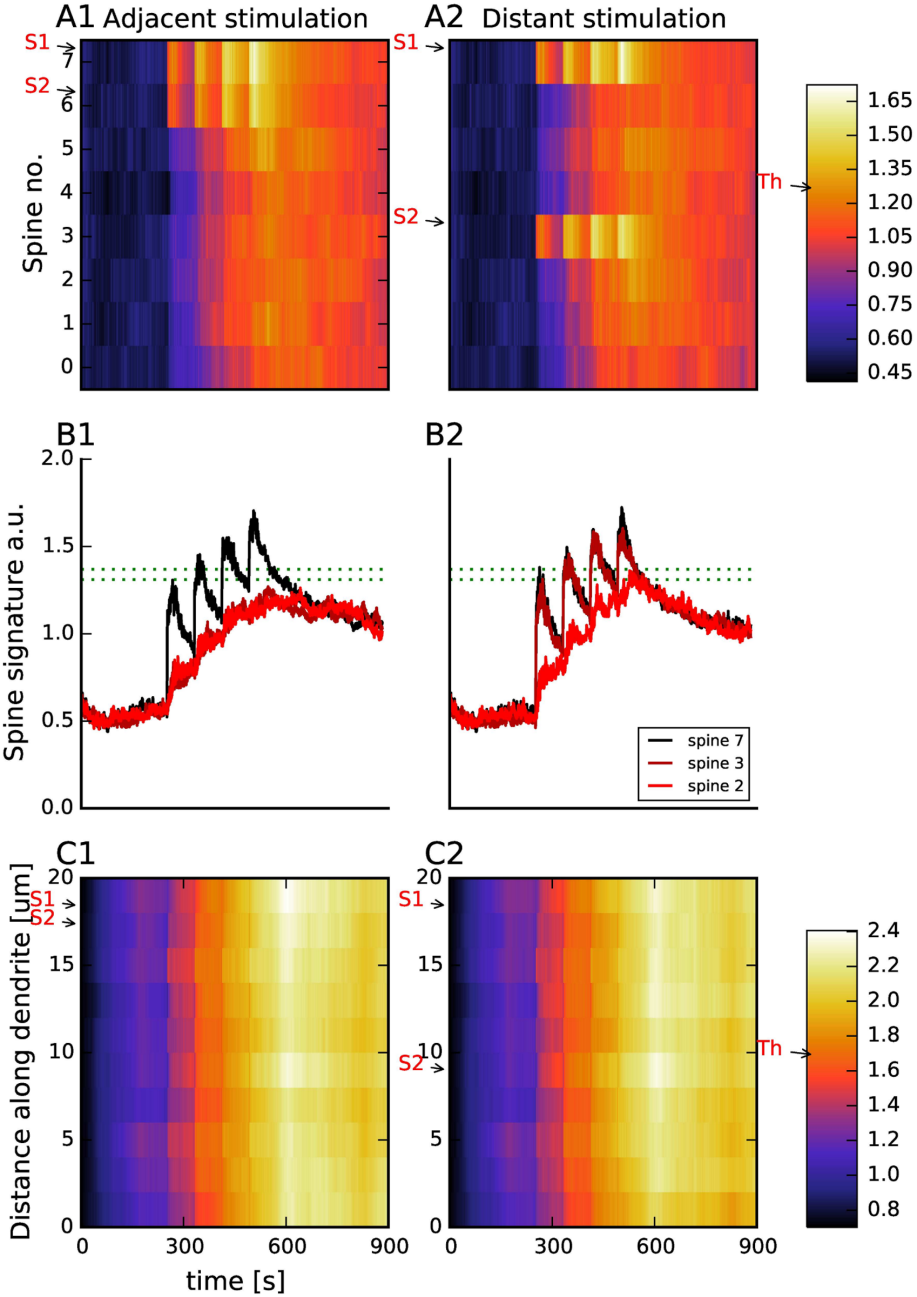
The spine signature exhibits spatial specificity, whereas the dendritic signature exceeds the threshold when two spines are stimulated, whether the spines are adjacent or separated. (A) A color plot of spine signature shows that only a few of the non-stimulated spines have a signature that exceeds the threshold. Arrow labeled Th shows threshold of the spine signature on the colorbar. A1: With stimulation of two adjacent spines, only the stimulated spines and an adjacent spine will exhibit LTP. A2: When separate spines are stimulated, the stimulated spines and also several nearby spines will exhibit LTP. Arrows labeled S1 and S2 show position of stimulated spines. (B) Time course of molecular signature for three spines (spine 2, 3 and 7), showing the difference in spine signature for stimulated and non-stimulated spines. (C) Molecular signatures in the dendrite. Arrow labeled Th shows threshold of the dendritic signature on the colorbar. Arrows labeled S1 and S2 show position of stimulated spines. B1: Spines 6 and 7 are stimulated. B2: Spines 3 and 7 are stimulated. Panels A1, A2, B1, B2, C1 and C2 show average traces. In these simulations we used initial conditions of 70% of PDE4 and ACs.

Both stimulation of separated and adjacent spines produce spine and dendritic signatures that exceed the threshold, and thus are able to induce L-LTP. Fig 11C shows that the dendritic signature exceeds the threshold throughout the dendritic branch. In contrast, Fig 11B reveals some degree of spatial specificity in the spine signature. Statistical analysis shows that for both adjacent and separated spine stimulation, molecular signatures of stimulated spines is greater than molecular signature of unstimulated spines (GLM, stimulus spacing and stimulation as factors, *F*(2,61) = 163, *F* >.0001; factor stimulation:*P* < 0.0001, factor spacing: *P* = 0.623. For both adjacent and separated spine stimulation, the duration of the spine signature above threshold of stimulated spines is significantly greater than the duration threshold 10 sec, (t-test, *T*(7) < 0.0001 for both adjacent and separated spine stimulation). In contrast, spine signatures of unstimulated spines are not above threshold for greater than 10 sec (t-test, *T*(7) = 0.9 for upper threshold, 0.06 for lower threshold for separated spine stimulation; *T*(7) = 0.79 for upper threshold, 0.016 for lower threshold for adjacent spine stimulation). For both adjacent and separated stimulation protocols, the CaMKII and Epac of the non-stimulated spines is lower than that of the stimulated spine, which is consistent with the gradients observed experimentally [76].

### Robustness of results

The ability to predict long-lasting forms of LTP does not depend on the precise details of the molecular signatures; instead the LTP predictions are similar for a range of thresholds, and for slight variations in the signature equations. The kinase-to-phosphatase balance, evaluated by molecular signatures, is thought to control direction of synaptic plasticity [36]. There are at least two ways of assessing this balance: either measuring the quantity of phosphorylated targets of kinases and phosphatases Eq (2), or assessing a ratio of kinase activity to phosphatase activity. Importantly, LTP predictions of our model are similar when the spine molecular signature evaluates the ratio of kinases (CaMKII and PKA) to phosphatases (PP1 and PP2B) (S7 Table).

The figures show a threshold range to demonstrate that the model makes the same predictions for any threshold value between the upper and lower thresholds, and does not require a precisely set threshold. To further assess robustness of our results, we evaluated individual simulations (realizations of protocols), that were executed with different random seeds. Note that the stochastic simulation includes a variation in injected quantity, which propagates (in some pathways with amplification) to yield as much as 30% variation in quantity of molecule activation. Tables 2 and 3 show that, despite variability in the time-course, the signatures for each realization of the long-lasting LTP eliciting protocols cross their thresholds for more than 10 sec uninterrupted. Further analysis (Tables 2 and 3) shows that these results are statistically significant. In addition increasing the time the spine signature remains over the threshold to 15 sec, does not significantly change the number of individual simulations that exceed the spine threshold (S8 Table).

**Table 2:** Robustness of the spine signature threshold. Standard error is an abbrevation for standard error of the mean. p value is significance of one-sided t-test comparing time signature is above the amplitude threshold to the 10 sec duration threshold. Degrees of freedom = 3 for t-tests of protocols with 4 different simulations, 7 for t-tests of protocols with 8 different simulations.

stimulation protocols	above the lower threshold			above the upper threshold		
	success /total	mean time ± standard error	p-value	success /total	mean time ± standard error	p-value
LFS	0/4	2.0±2.0	0.9849	0/4	0.0±0.0	1
ISO	0/4	2.0±1.0	0.9986	0/4	0.0±0.0	1
HFS	4/4	77.0±14.0	0.0092	4/4	50.0±4.0	0.0012
4xHFS-3s	8/8	202.0±21.0	0.0001	8/8	149.0±15.0	0.0001
4xHFS-80s	8/8	463.0±25.0	0.0001	8/8	376.0±20.0	0.0001
ISO+HFS	4/4	131.0±26.0	0.0094	4/4	95.0±14.0	0.0047
ISO+LFS	4/4	119.0±20.0	0.0060	4/4	53.0±14.0	0.0260
HFS no PKA	0/4	3.0±2.0	0.9906	0/4	1.0±1.0	0.9993
ISO+HFS no PKA	8/8	26.0±1.0	0.0001	8/8	20.0±1.0	0.0001
4xHFS-80s no PKA	8/8	94.0±6.0	0.0007	8/8	58.0±4.0	0.0010
4xHFS-3s no PKA	4/4	51.0±3.0	0.0004	4/4	42.0±2.0	0.0001
ISO+LFS no PKA	0/4	0.0±0.0	1	0/4	0.0±0.0	1
Propranolol+4xHFS	8/8	337.0±13.0	0.0001	8/8	270.0±19.0	0.0001
ICI-118551+4xHFS	4/4	327.0±5.0	0.0001	4/4	232.0±10.0	0.0001
Carvedilol+HFS	4/4	20.0±4.0	0.0427	0/4	5.0±1.0	0.9892
Carvedilol+LFS	0/4	0.0±0.0	1	0/4	0.0±0.0	1
Carvedilol+2xHFS	4/4	180.0±31.0	0.0057	4/4	119.0±22.0	0.0082
Carvedilol+3xHFS	4/4	274.0±23.0	0.0007	4/4	193.0±18.0	0.0011

**Table 3:** Robustness of the dendritic signature threshold. Standard error is an abbrevation for standard error of the mean. p value is significance of one-sided t-test comparing time signature is above the amplitude threshold to the 10s duration threshold. Degrees of freedom = 3 for t-tests of protocols with 4 different simulations, 7 for t-tests of protocols with 8 different simulations.

stimulation protocols	above the lower threshold			above the upper threshold		
	success /total	mean time ± standard error	p-value	success /total	mean time ± standard error	p-value
LFS	0/4	0.0±0.0	1	0/4	0.0±0.0	1
ISO	0/4	0.0±0.0	1	0/4	0.0±0.0	1
HFS	0/4	0.0±0.0	1	0/4	0.0±0.0	1
4xHFS-3s	8/8	208.0±52.0	0.0032	8/8	167.0±51.0	0.0089
4xHFS-80s	8/8	261.0±39.0	0.0002	8/8	173.0±45.0	0.0044
ISO+HFS	4/4	389.0±2.0	0.0001	4/4	386.0±4.0	0.0001
ISO+LFS	4/4	314.0±5.0	0.0001	4/4	309.0±5.0	0.0001
HFS no PKA	0/4	0.0±0.0	1	0/4	0.0±0.0	1
ISO+HFS no PKA	8/8	16.0±1.0	0.0001	8/8	14.0±1.0	0.0011
4xHFS-80s no PKA	2/8	7.0±2.0	0.9017	1/8	3.0±2.0	0.9980
4xHFS-3s no PKA	4/4	31.0±1.0	0.0001	4/4	27.0±0.0	0.0001
ISO+LFS no PKA	0/4	0.0±0.0	1	0/4	0.0±0.0	1
Propranolol+4xHFS	8/8	127.0±15.0	0.0001	8/8	77.0±22.0	0.0103
ICI-118551+4xHFS	0/4	1.0±1.0	0.9951	0/4	0.0±0.0	1
Carvedilol+HFS	0/4	0.0±0.0	1	0/4	0.0±0.0	1
Carvedilol+LFS	0/4	0.0±0.0	1	0/4	0.0±0.0	1
Carvedilol+2xHFS	3/4	62.0±23.0	0.0548	3/4	15.0±8.0	0.2931
Carvedilol+3xHFS	4/4	264.0±14.0	0.0002	4/4	202.0±15.0	0.0005

**Fig 12:**
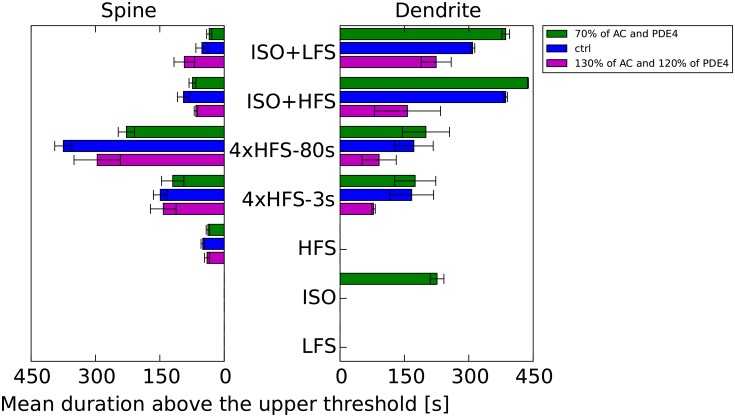
Time spent by spine or dendritic signatures above their respective thresholds for control protocols. Comparison between model with 70% of AC and PDE4 concentration of the control model, control model, and model with 130% of AC and 120% of PDE4.

To further evaluate robustness of the results, we repeated simulations with variations of two sets of parameters. The first set of parameter variations lowered both AC and PDE4 concentration by 30%. The second set of parameter variations increased AC concentration by 30% and PDE4 concentration by 20%. In both cases, AC and PDE4 quantities were varied together to maintain a 30 nM basal cAMP concentration. Fig 12 shows the mean duration that the spine or dendritic signatures remained above their respective thresholds. Though the signatures varied significantly with parameter variation and trial (as shown by the standard error of the mean), in all cases both signatures were exceeded only for those stimulation protocols that experimentally yield LTP. It is also worth noting that simulations of models with higher AC levels were more noisy because of competition for calmodulin.

## Discussion

To predict long-lasting forms of LTP we developed a stochastic reaction-diffusion model of a dendrite with spines. We looked at activity of the key molecular species during the first 10 min following plasticity induction, because long-lasting LTP is blocked by protein kinase inhibitors applied during or immediately after induction of LTP [57, 77]. A relatively short duration above the threshold is in agreement with [64], showing that temporal window of CaMKII activation required for synaptic plasticity and learning is narrow. We devised a set of molecular signatures: one in the spine and one in the dendrite, that predict induction of long-lasting forms of LTP. We demonstrated that two molecular signatures can explain the results of a large number of experimental protocols. Additional simulations suggested the complex role of the *β*AR activation in long-lasting forms of LTP. The spatial aspect of these simulations was critical, as a single molecular signature that calculated a spatial average of molecular activity was unable to predict the induction of all forms of long-lasting forms of LTP. Fig 12 clearly shows that the relationship between the dendritic signature and the spine signature depends on the stimulation protocol.

Separate molecular signatures in the spine and in the dendrite represent distinct phenomena. Two signatures can be viewed as corresponding to synaptic tagging and capture [63, 65], a theory explaining how signaling molecules in different spatial compartments play different roles in L-LTP. Synaptic tagging involves labeling of specific dendritic spines that are to undergo long term plasticity, and capture implies that a spatially non-specific signal induces synthesis of plasticity related proteins (PRPs), and in some cases, initiates transcription [78]. PRPs are synthesized locally or trafficked up the dendrite and captured by tagged spines to stabilize synaptic strength. Crossing the threshold by the spine molecular signature can be viewed as setting the tag and crossing the threshold by the dendritic molecular signature corresponds to sending the signal initiating the synthesis of PRPs.

**Fig 13:**
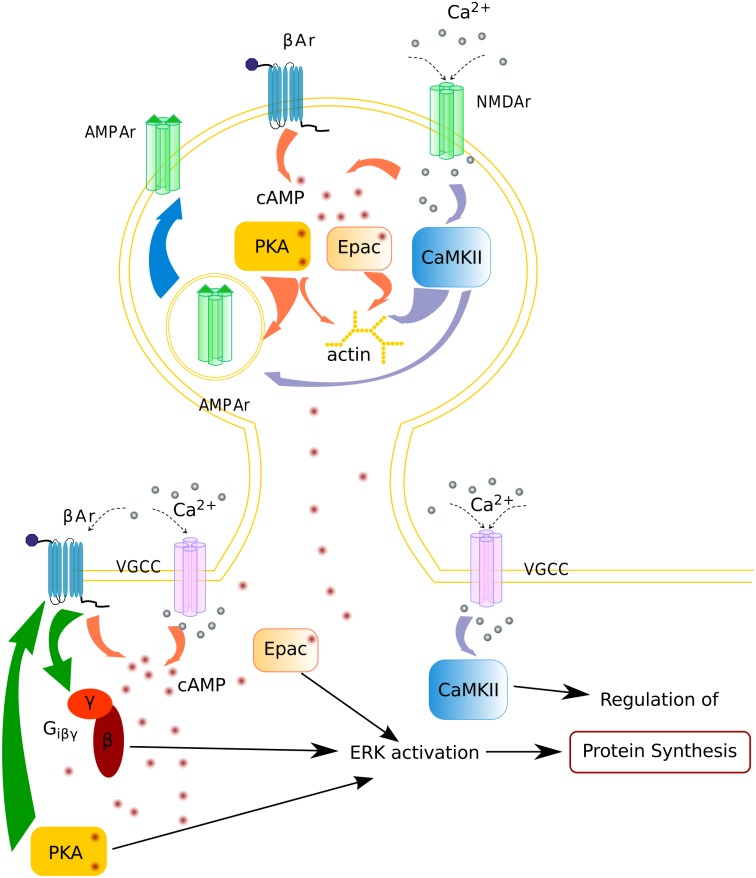
Schematic diagram depicting core mechanisms underlying stabilization of the synaptic strength that accompanies long-term synaptic plasticity. In the dendrite *β*AR activation is required for protocols with low calcium influx, e.g. LFS. In the dendrite *β*AR activation either by G_s_ coupling or by switching to G_i_ coupling is needed for dendrite specific changes.

In constructing the spine molecular signature, we evaluated molecules that are implicated in synaptic tagging, AMPA receptor insertion, actin remodeling and structural plasticity [72, 79–82] (Fig 13). Blocking CaMKII activity [61–63] has been shown to block tagging, and CaMKII also is implicated in the actin remodeling underlying structural plasticity [83–85] by triggering SynGAP dispersion from synaptic spines [86]. PKA is required for synaptic tagging [56, 66, 87, 88] and is implicated in structural plasticity. PKA modulates the activity of LIM kinase [89, 90], which phosphorylates (and inhibits) cofilin allowing for actin polymerization. Cofilin-mediated actin dynamics regulates spine morphology and AMPAR trafficking during synaptic plasticity [91, 92]. Epac anchors in the PSD [93] and triggers changes to spine cytoskeleton via Rap1 activation [94]. Interestingly, synapses stimulated by HFS while blocking PKA activity fail to be tagged [88], whereas ISO+HFS stimulation while blocking PKA still yields L-LTP [14]. Our simulations suggest that this seemingly contradictory result arises from the difference between the amount of Epac provided by HFS alone versus ISO+HFS. The plausibility of the spine signature is evident from its time course, which is comparable to the dynamics of molecular activation measured using live cell imaging [80].

The molecular signature in the dendrite takes into account molecules that play a role in synthesis of PRPs (Fig 13). Both PKA and Epac activate ERK via Rap1 regulation [95–98]. Also, PKA phosphorylation of *β*2AR can produce ERK activation by switching the *β*2AR coupling from G_s_ to G_i_ [15–17], though this has not been directly demonstrated in neurons. ERK has been shown to be critical in L-LTP [12, 13, 55, 99–101] and the synthesis of PRPs [61]. Both PKA and ERK can phosphorylate CREB, a molecule directly implicated in transcription. CaMKII is required for regulation of protein synthesis via phosphorylation of cytoplasmic polyadenylation element binding protein [102, 103] in hippocampal plasticity, but see [61, 62]. Though both spine and dendritic signatures incorporated the same molecules, they have different downstream targets in the spine and in the dendrite. Thus the two molecular signatures set the stage for future models that incorporate control of actin dynamics in the spine and ERK activation in the dendrite.

Several other models have evaluated molecular dependence and temporal sensitivity of L-LTP induction. The most comprehensive model of signaling pathways leading to transcription of mRNA [104] demonstrated that different temporal stimulation patterns could recruit different mRNAs. In agreement with their results, our simulations showed that different stimulation patterns produced different patterns of elevation of various kinases. It would be quite interesting to couple our dendritic model to downstream modules of the model presented in [104] to evaluate control of transcription by L-LTP stimulation patterns. Several other models investigated synaptic tagging and capture [105–107] at hippocampal CA3-CA1 synapses. All of these models were able to predict various aspects of the synaptic tagging and capture hypothesis. Nonetheless, these models used simplified and abstract equations for activation of key kinases and phosphatases; thus it is not clear how well they could extrapolate to alternative stimulation patterns. Another model [108] also used streamlined equations for activation of key kinases and phosphatases, but included a model of histone deacetylation, which regulates transcription [109]. That model suggested that promoting histone acetylation while simultaneously slowing cAMP degradation could help in restoring L-LTP, which is impaired in mouse models of Rubinstein-Taybi syndrome, a condition resulting in lower levels of CREB binding protein, which reduces transcription.

Our simulations of a dendrite with multiple spines are consistent with the spatial specificity of homo- and heterosynaptic plasticity suggested by imaging of spine morphological plasticity. Stimulation of two spines on the same branch produces a dendritic signature that crosses the threshold along the entire branch, regardless of the spatial configuration of those stimulated spines. This result is consistent with [75], showing that one train of 5 Hz stimulation applied to two spines on the same branch saturates ERK activation in that branch. During these simulations, spine signatures of the unstimulated spines are elevated, although lower than those of the stimulated spines. This observation is consistent with the gradients observed experimentally [76]. Furthermore, the increase in signature of non-stimulated spines is consistent with the observation of a reduced LTP threshold heterosynaptically [73]. It is, however, also possible that not all spines will exhibit potentiation due to competition for resources, as in [74]. Our model does not take into account this competition, but such a model would allow only the spines with the highest signatures to capture PRPs, and thus non-stimulated spines with lower signatures would not exhibit LTP. The agreement between these simulations and experiments suggests the model could be used to predict the spatial pattern of LTP in response to in vivo like stimulation patterns.

We evaluated AMPAR phosphorylation by CaMKII and PKA as an indicator of E-LTP, and found agreement between our simulations and experimental results [70, 110, 111]. The brief duration of the AMPAR phosphorylation in our model is likely due to absence of AMPAR re-cycling mechanisms [112]. Previous work has shown AMPAR recycling contributes to bistability [113], and insertion of a phosphorylated AMPAR may protect it from dephosphorylation. Alternatively, AMPAR phosphorylation may only be a trigger for insertion, and the time course of E-LTP may reflect the removal of AMPARs in the synapse.

Induction of long-lasting LTP initiates a cascade of complex molecular interactions; therefore signaling pathway modeling is a useful approach to facilitate understanding of this complexity. In addition to confirming the plasticity outcome and molecular dependence for numerous LTP induction protocols, our model makes several experimentally testable predictions. Our model suggests that *β*AR signaling through non-conventional pathways is necessary in the dendrite, therefore ICI-118,551, a complete *β*AR antagonist, will likely block long-lasting LTP induced with 4xHFS-80s, a model prediction that needs to be tested experimentally. Moreover, the model suggests that both conventional (G_s_-activated) and non-conventional (G_i_-activated) pathways are required for ISO+LFS and ISO+HFS to produce long-lasting LTP, therefore we predict that bath application of carvedilol, which blocks norepinephrine binding but allows G_i_ recruitment, will not induce long-lasting LTP. Simulations of bath application of carvedilol followed by one, two and three trains of HFS shows that high enough calcium might substitute for G_s_ activation in L-LTP induction, but that both G_s_ and G_i_ might be necessary for L-LTP induction using LFS. Though our model focuses on *β*AR signaling, CA1 neurons express dopamine receptors, which have been implicated in some forms of long-lasting LTP [114]. If such receptors are shown to undergo switching of G_s_ to G_i_ coupling, then these receptors also may contribute to a plethora of long-lasting forms of LTP. In summary, our model suggests that the non-linearity of signaling pathway interactions may explain why experimentally blocking any of the molecules included in our signature can disrupt long-lasting LTP.

## Supporting information

S1 Table Parameters of the signaling pathways. * Rapid dissociation after enzyme reaction prevents accumulation of these intermediate forms. ** CaMKII phosphorylation reactions involving Complex are required to produce the observed calcium sensitivity, and capture the probability that two calmodulin bound CaMKII subunits are adjacent in the holoenzyme. Abbreviations: NE—norepinephrine, G_*βγ*_—*βγ* subunit of G protein, PMCA—plasma membrane Ca^2+^ ATPase, ncx—Na^+^/Ca^2+^ exchanger, pCaMKII—Thr 286 phosphorylated CaMKII, PKAc—catalytic subunit of PKA, PKAr—regulatory subunit of PKA, Ip35—Thr35 phosphorylated I1, PP1—protein phosphatase 1, PDE4—phosphodiesterase 4, GluR1—glutamate receptor 1, pS831GluR1—Ser831 phosphorylated GluR1, pS845GluR1—Ser845 phosphorylated GluR1.
(PDF)

S2 Table Diffusion rates of diffusible species. Abbreviations same as in S1 Table.
(PDF)

S3 Table Initial conditions of cytosolic species. Initial conditions of remaining cytosolic species (Complex, pComplex, I1PKAc, PKAc-PDE4-cAMP, CaBCa, PDE1CaMCa_4_cAMP, Ip35PP2BCaMCa_4_, L, pPDE4-cAMP, PKAc, CaB) were set to 0.
(PDF)

S4 Table Initial conditions of species anchored in the spine head and in the dendrite membrane. Initial conditions of remaining anchored species (LRG_s_, *LRG*_*sβγ*_, pR, pLR, ppLR, pppLR, ppppLR, pR, ppR, pppR, ppppR, PKAcR, PKAcpR, PKAcppR, PKAcpppR, PKAcLR, PKAcpLR, PKAcppLR, PKAcpppLR, ppppLRG_i_, ppppRG_i_, ppppRG_*iβγ*_, ppppLRG_*iβγ*_, G_*iβγ*_, G_*αi*_ GDP, AC1G_*α*s_ GTPG_*αi*_ GTPCaMCa_4_, AC1G_*α*s_ GTPG_*αi*_ GTP, G_*αi*_ GTP AC1G_*αi*_ GTPCaMCa_4_, AC1GsGiCaMCa_4_ATP, AC1G_*α*s_ GTPCaMCa_4_, AC1G_*αi*_ GTP, AC1G_*α*s_ GTPCaMCa_4_ ATP AC1G_*αi*_ GTPCaMCa_4_ ATP,) were set to 0 both in the spinehead and in the dedritic submembrane.
(PDF)

S5 Table Initial conditions of species anchored in the PSD. Initial conditions of remaining anchored species (Leak, ncx, ncxCa, pmca, pmcaCa, GluR1-CKCam, GluR1-CKp, GluR1-CKpCaM, GluR1-PKAc, pS831GluR1, pS831-GluR1-PKAc, pS831GluR1-PP1, pS845GluR1, pS845GluR1-CKCam, pS845GluR1-CKp, pS845GluR1-CKpCaM, pS845GluR1-PP1, pS845GluR1-PP2B, pS845pS831GluR1, pS845pS831GluR1-PP1, pS845pS831GluR1-PP2B).
(PDF)

S6 Table Parameters of the norepinephrine release model fitted to Eq (1). (PDF)

S7 Table Robustness of the spine signature. The kinase-to-phosphatase balance, evaluated by molecular signatures, is thought to control direction of synaptic plasticity. There are at least two ways of assessing this balance: either measuring the quantity of phosphorylated targets of kinases and phosphatases, or assessing a ratio of kinase activity to phosphatase activity. In this table spine molecular signature has an alternative form and evaluates Epac activity and the ratio of active CaMKII and active PKA to active phosphatases (PP1 and PP2B). This form of spine signature is very noisy, hence to induce spine specific changes, the spine signature has to exceed its threshold for 10 sec uninterrupted.
(PDF)

S8 Table Robustness of the spine signature threshold. (PDF)
