# Supplementary material for: β-adrenergic signaling broadly contributes to LTP induction
Source: PLoS Comput Biol. 2017 Jul 24;13(7):e1005657. doi: 10.1371/journal.pcbi.1005657 (PMC5546712; doi:10.1371/journal.pcbi.1005657)
Supplement: S1 Table — * Rapid dissociation after enzyme reaction prevents accumulation of these intermediate forms. ** CaMKII phosphorylation reactions involving Complex are required to produce the observed calcium sensitivity, and capture the probability that two calmodulin bound CaMKII subunits are adjacent in the holoenzyme. Abbreviations: NE—norepinephrine, Gβγ—βγ subunit of G protein, PMCA—plasma membrane Ca2+ ATPase, ncx—Na+/Ca2+ exchanger, pCaMKII—Thr 286 phosphorylated CaMKII, PKAc—catalytic subunit of PKA, PKAr—regulatory subunit of PKA, Ip35—Thr35 phosphorylated I1, PP1—protein phosphatase 1, PDE4—phosphodiesterase 4, GluR1—glutamate receptor 1, pS831GluR1—Ser831 phosphorylated GluR1, pS845GluR1—Ser845 phosphorylated GluR1. (PDF) [file pcbi.1005657.s001.pdf]

Table S1: **Parameters of the signaling pathways.** * Rapid dissociation after enzyme reaction prevents accumulation of these intermediate forms. ** CaMKII phosphorylation reactions involving Complex are required to produce the observed calcium sensitivity, and capture the probability that two calmodulin bound CaMKII subunits are adjacent in the holoenzyme. Abbreviations: NE – norepinephrine, $G_{\beta\gamma}$ – $\beta\gamma$ subunit of G protein, PMCA – plasma membrane Ca^{2+} ATPase, ncx – Na^{+}/Ca^{2+} exchanger, pCaMKII – Thr 286 phosphorylated CaMKII, PKAc – catalytic subunit of PKA, PKAr – regulatory subunit of PKA, Ip35 – Thr35 phosphorylated I1, PP1 – protein phosphatase 1, PDE4 – phosphodiestrase 4, GluR1 – glutamate receptor 1, pS831GLuR1 – Ser831 phosphorylated GLuR1, pS845GLuR1 – Ser845 phosphorylated GluR1.

Reaction	$\mathbf{k_f}$ $\left[\frac{1}{\mathrm{nM\,ms}}\right]$	$\mathbf{k_b}$ $\left[\frac{1}{\mathrm{ms}}\right]$	$\mathbf{k_{cat}}$ $\left[\frac{1}{\mathrm{ms}}\right]$	source
$\mathrm{PMCA + Ca \rightleftharpoons PMCACa \rightarrow PMCA + Ca_{out}}$	5.00E-5	7.00E-3	3.50E-3	[115]
$\mathrm{ncx + Ca \rightleftharpoons ncxCa \rightarrow ncx + Ca_{out}}$	1.68E-5	1.12E-2	5.60E-3	[116, 117]
$\mathrm{Ca_{Out} + Leak \rightleftharpoons Ca_{Out}Leak \rightarrow Ca + Leak}$	1.50E-6	1.10E-3	1.10E-3	adjusted to maintain basal calcium concentration of 70 nM
$\mathrm{Ca + Calbindin \rightleftharpoons CalbindinCa}$	2.80E-5	1.96E-2		[118]
$\mathrm{Ca + Buffer \rightarrow CaBuffer}$	2.80E-5			**
$\mathrm{2Ca + CaM \rightleftharpoons CaMCa_2}$	6.00E-6	9.10E-3		[119]
$\mathrm{2Ca + CaMCa_2 \rightleftharpoons CaMCa_4}$	1.00E-4	1.00		[120]
$\mathrm{Ng + CaM \rightleftharpoons CaMCa_4}$	2.80E-5	3.60E-2		[21]
$\mathrm{PP2B + CaM \rightleftharpoons PP2BCaM}$	4.60E-6	1.20E-6		PMR
$\mathrm{PP2B + CaMCa_2 \rightleftharpoons PP2BCaM_2}$	4.60E-6	1.20E-7		PMR
$\mathrm{PP2B + CaMCa_4 \rightleftharpoons PP2BCaM_4}$	4.60E-5	1.20E-6		[121]
$\mathrm{PP2BCaM + 2Ca \rightleftharpoons PP2BCaM_2}$	6.00E-4	9.1E-2		[122]
$\mathrm{PP2BCaM_2 + 2Ca \rightleftharpoons PP2BCaM_4}$	1.00E-4	1		[123]
$\mathrm{CaMKII + CaMCa_4 \rightleftharpoons CaMKIICaMCa_4}$	1.00E-5	3.00E-3		[124]
$\mathrm{CaMKIICaMCa_4 + CaMKIICaMCa_4 \rightleftharpoons Complex}$	1.00E-7	1.00E-2		**
$\mathrm{pCaMKIICaMCa_4 + CaMKIICaMCa_4 \rightleftharpoons pComplex}$	1.00E-7	1.00E-2		**
$\mathrm{X + Complex \rightleftharpoons X + pComplex}$<br>$\mathrm{X = \{CaMKIICaMCa_4, pCaMKIICaMCa_4\}}$	1.00E-7			**
$\mathrm{Complex + Complex \rightleftharpoons Complex + pComplex}$	1.00E-5			**
$\mathrm{Complex + pComplex \rightleftharpoons pComplex + pComplex}$	3.00E-5			**
$\mathrm{pCaMKIICaMCa4 \rightleftharpoons CaMCa4 + pCaMKII}$	8.00E-7	1.00E-5		[124]

$pX + PP1 \rightleftharpoons XPP1 \rightarrow X + PP1$<br>$X = \{CaMKII, CaMKIICaMCa4\}$	4.00E-9	3.40E-4	8.60E-5	[125, 126, 10]
$NE \rightleftharpoons NEBuffer$	5.00E-4	2.00E-9		adjusted to maintain basal 10 nM of NE
$NE + \beta2AR \rightleftharpoons NE\beta2AR$	5.56E-6	5.00E-3		[127, 128]
$NE\beta2AR + G_s \rightleftharpoons NE\beta2ARG_s \rightarrow NE\beta2ARG_{s\beta\gamma}$<br>$+G_{s\alpha}GTP$	6.00E-7	1.00E-6	2.00E-2	[129]
$\beta2AR + G_s \rightleftharpoons \beta2ARG_s$	4.00E-8	3.00E-7		PMR
$NE + \beta2ARG_s \rightleftharpoons NE\beta2ARG_s \rightarrow NE\beta2ARG_{s\beta\gamma}$<br>$+G_{s\alpha}GTP$	2.50E-6	5.00E-4	2.00E-2	[127, 128]
$NE\beta2ARG_{s\beta\gamma} \rightarrow NE\beta2AR + G_{s\beta\gamma}$	8.00E-2			*
$G_{s\alpha}GTP \rightarrow G_{s\alpha}GDP$	1.00E-2			[130, 131]
$G_{s\alpha}GTP + G_{s\beta\gamma} \rightarrow G_s$	1.00E-1			[132]
$X \rightleftharpoons XBuffer,$<br>$X = \{Carvedilol, propranolol, ICI-118,551\},$	5.00E-1	2.00E-9		adjusted to mimic experimental conditions
$Carvedilol + \beta2AR \rightarrow Carvedilol\beta2AR$	6.08E-5	7.67e-6		[133]
$Carvedilol\beta2AR + G_i \rightarrow Carvedilol\beta2ARG_i$	6e-8	0.3e-3		[18]
$ICI-118,551 + \beta2AR \rightarrow ICI-118,551\beta2AR$	1.27e-5	3.5e-6		[133]
$Propranolol + \beta2AR \rightarrow Propranolol\beta2AR$	6.05E-5	7.67E-6		[133]
$Propranolol\beta2AR + G_i \rightarrow Propranolol\beta2ARG_i$	6.00E-8	1.2E-3		[18]
$X + PKAc \rightleftharpoons XPKAc \rightarrow pX + PKAc,$<br>$X = \{NE\beta2AR, pNE\beta2AR\}$	8E-7	4.48E-3	1.00E-3	adjusted to effect
$ppNE\beta2AR + PKAc \rightleftharpoons ppNE\beta2ARPKAc \rightarrow$<br>$pppNE\beta2AR + PKAc$	1.712E-5	4.48E-3	1.00E-3	adjusted to effect
$pppNE\beta2AR + PKAc \rightleftharpoons pppNE\beta2ARPKAc \rightarrow$<br>$ppppNE\beta2AR+PKAc$	1.712E-3	4.48E-3	1.00E-3	adjusted to effect
$ppppNE\beta2AR + G_i \rightleftharpoons ppppNE\beta2ARG_i \rightarrow$<br>$ppppNE\beta2ARG_{i\beta\gamma} + G_{i\alpha}GTP$	1.50E-4	2.50E-4	1.25E-4	adjusted to effect
$ppppX + G_{i\beta\gamma} \rightarrow ppppXG_{i\beta\gamma}$<br>$X = \{\beta2AR, NE\beta2AR\}$	1.00E-3			adjusted to effect
$\beta2AR + PKAc \rightleftharpoons \beta2ARPKAc \rightarrow$<br>$p\beta2AR + PKAc$	4.00E-8	4.48E-3	1.00E-3	adjusted to effect
$p\beta2AR + PKAc \rightleftharpoons p\beta2ARPKAc \rightarrow$<br>$pp\beta2AR + PKAc$	4.00E-7	4.48E-3	1.00E-3	adjusted to effect

$pp\beta 2AR + PKAc \rightleftharpoons pp\beta 2ARPKAc \rightarrow ppp\beta 2AR + PKAc$	4.00E-6	4.48E-3	1.00E-3	adjusted to effect
$ppp\beta 2AR + PKAc \rightleftharpoons ppp\beta 2ARPKAc \rightarrow pppp\beta 2AR + PKAc$	4.00E-4	4.48E-3	1.00E-3	adjusted to effect
$pppp\beta 2AR + G_i \rightleftharpoons pppp\beta 2ARG_i \rightarrow pppp\beta 2ARG_{i\beta\gamma}+G_{i\alpha}GTP$	7.50E-5	1.25E-4	6.25E-5	adjusted to effect
$pX \rightarrow X$ $X = \{pppNE\beta 2AR, ppNE\beta 2AR, pNE\beta 2AR, NE\beta 2AR, ppp\beta 2AR, pp\beta 2AR, p\beta 2AR, \beta 2AR\}$	2.50E-6			[134]
$G_{i\alpha}GTP \rightarrow G_{i\alpha}GDP$	1.25E-4			[131]
$G_{i\alpha} + G_{i\beta\gamma} \rightarrow G_i$	1.25E-3			[132]
$AC1 + G_{s\alpha}GTP \rightleftharpoons AC1G_{s\alpha}GTP$	3.85E-5	1.00E-2		[135]
$AC1 + CaMCa_4 \rightleftharpoons AC1CaMCa_4$	6.00E-6	9.00E-4		[136]
$AC1G_{s\alpha}GTP + CaMCa_4 \rightleftharpoons AC1G_{s\alpha}GTPCaMCa_4$	6.00E-6	9.00E-4		[25]
$AC1CaMCa_4 + ATP \rightleftharpoons AC1CaMCa_4ATP \rightarrow AC1CaMCa_4 + cAMP$	1.00E-5	2.27	5.68E-3	[25]
$AC1G_{s\alpha}GTPCaMCa_4 + ATP \rightleftharpoons AC1G_{s\alpha}GTPCaMCa_4\,ATP \rightarrow AC1G_{s\alpha}GTPCaMCa_4 + cAMP$	1.00E-5	2.27	2.84E-2	[25, 137]
$AC1CaMCa_4 + G_{i\alpha}GTP \rightleftharpoons AC1CaMCa_4G_{i\alpha}GTP$	6.25E-5	1.00E-2		[138, 139]
$AC1G_{s\alpha}GTP + G_{i\alpha}GTP \rightleftharpoons AC1G_{s\alpha}GTPG_{i\alpha}GTP$	6.25E-5	1.00E-2		[138, 139]
$AC1G_{i\alpha}GTP + G_{s\alpha}GTP \rightleftharpoons AC1G_{i\alpha}GTPG_{s\alpha}GTP$	6.25E-5	1.00E-2		[138, 139]
$AC1G_{s\alpha}GTPG_{i\alpha}GTP + CaMCa_4 \rightleftharpoons AC1G_{s\alpha}GTPG_{i\alpha}GTPCaMCa_4$	6.00E-6	9.00E-4		[138]
$AC1G_{i\alpha}GTPCaMCa_4 + ATP \rightleftharpoons AC1G_{i\alpha}GTPCaMCa_4\,ATP \rightarrow AC1G_{i\alpha}GTPCaMCa_4 + cAMP$	1.00E-5	2.27	5.684E-4	[138]
$AC1G_{i\alpha}GTPG_{s\alpha}GTPCaMCa_4 + ATP \rightleftharpoons AC1G_{i\alpha}GTPG_{s\alpha}GTPCaMCa_4\,ATP \rightarrow AC1G_{i\alpha}GTPG_{s\alpha}GTPCaMCa_4 + cAMP$	1.00E-5	2.27	2.84E-3	[138]
$AC8 + CaMCa_4 \rightleftharpoons AC8CaMCa_4$	1.25E-6	1.00E-3		[136]
$AC8CaMCa_4 + ATP \rightleftharpoons AC8CaMCa_4ATP \rightarrow AC8CaMCa_4 + cAMP$	1.00E-5	2.27	2.84E-3	[140]
$Epac2 + cAMP \rightleftharpoons Epac2 - cAMP$	3.10E-8	6.51E-5		[141]
$PKA + 2cAMP \rightleftharpoons PKAcAMP_2$	2.61E-7	6.00E-5		[142, 143]
$PKAcAMP_2 + 2cAMP \rightleftharpoons PKAcAMP_4$	3.46E-7	6.00E-4		[144, 143]
$PKAcAMP_4 \rightarrow PKAr + 2xPKAc$	4.80E-5	5.10E-6		[145]
$I1 + PKAc \rightleftharpoons I1PKAc \rightarrow Ip35 + PKAc$	1.40E-6	5.60E-3	1.40E-3	[146]

$Ip35 + PP1 \rightleftharpoons Ip35PP1$	1.00E-6	1.10E-6		[147, 148]
$Ip35 + PP2B \rightleftharpoons Ip35PP2B \rightarrow I1 + PP2B$	9.62E-5	0.33	5.5E-2	[146, 149]
$Ip35PP1 + PP2B \rightleftharpoons Ip35PP1PP2B \rightarrow I1 + PP1PP2B$	9.62E-5	0.33	5.5E-2	[146, 149]
$PP1PP2B \rightarrow PP1 + PP2B$	5.50E-2			*
$AMP \rightarrow ATP$	1.00E-3			
$PDE4 + cAMP \rightleftharpoons PDE4cAMP \rightarrow PDE4 + AMP$	2.17E-5	6.90E-2	1.72E-2	[150]
$PDE4 + PKAc \rightleftharpoons PDE4PKAc \rightarrow pPDE4 + PKAc$	4.28E-7	5.60E-4	1.25E-4	[68]
$pPDE4 + cAMP \rightleftharpoons pPDE4cAMP \rightarrow pPDE4 + AMP$	4.33E-4	1.38	3.45E-1	[68]
$PDE4cAMP + PKAc \rightleftharpoons PDE4cAMPPKAc \rightarrow pPDE4cAMP + PKAc$	4.28E-7	5.60E-4	1.25E-4	[68]
$pPDE4 \rightarrow PDE4$	2.50E-6			adjusted to maintain basal cAMP concentration between 30 nM and 50 nM
$PDE1B + CaMCa_4 \rightleftharpoons PDE1BCaMCa4$	1.00E-4	1.00E-3		[151]
$PDE1BCaMCa_4 + cAMP \rightleftharpoons PDE1BCaMCa4cAMP \rightarrow PDE1BCaMCa_4 + AMP$	4.60E-6	4.40E-2	1.10E-2	[152]
$X + PKAc \rightleftharpoons XPKAc \rightarrow pS845X + PKAc$, $X = GluR1, pS831GluR1$	4.00E-6	2.40E-2	6.00E-3	[113, 20]
$X + Y \rightleftharpoons XY \rightarrow pS831X + Y$ $X = \{GluR1, pS845GluR1\}$, $Y = \{pCaMKII, CaMKIICaMCa_4\}$	2.22E-8	1.60E-3	4.00E-4	[113, 20]
$X + pCaMKIICaMCa_4 \rightleftharpoons XpCaMKIICaMCa_4 \rightarrow pS831X + pCaMKIICaMCa_4$, $X = \{GluR1, pS845GluR1\}$	2.78E-8	2.00E-3	5.00E-4	[113, 20]
$pS831GluR1 + PP1 \rightleftharpoons pS831GluR1PP1 \rightarrow GluR1 + PP1$	8.75E-7	1.4E-3	3.5E-4	[20], affinity from [113]
$pS845GluR1 + PP1 \rightleftharpoons pS845GluR1PP1 \rightarrow GluR1 + PP1$	8.7E-7	6.80E-4	1.7E-4	[20], affinity [113]
$pS845pS845GluR1 + PP1 \rightleftharpoons pS845pS845GluR1PP1 \rightarrow X + PP1,\ X = \{pS845GluR1, pS831GluR1\}$	8.75E-7	1.40E-3	3.50E-4	[20], affinity [113]

$\mathrm{pS845X} + \mathrm{PP2BCaMCa_4} \rightleftharpoons \mathrm{pS845XPP2B} \rightarrow$ $\mathrm{X} + \mathrm{PP2BCaMCa_4}$, $\mathrm{X} = \{\mathrm{GluR1}, \mathrm{pS831GluR1}\}$	2.01E-6	8.00E-3	2.00E-3	[113, 20]

References

[1] Morris RG, Anderson E, Lynch GS, Baudry M. Selective impairment of learning and blockade of long-term potentiation by an N-methyl-D-aspartate receptor antagonist, AP5. Nature. 1986;319(6056):774–776.

[2] Tsien JZ, Huerta PT, Tonegawa S. The essential role of hippocampal CA1 NMDA receptor–dependent synaptic plasticity in spatial memory. Cell. 1996;87(7):1327–1338.

[3] Shimizu E, Tang YP, Rampon C, Tsien JZ. NMDA receptor-dependent synaptic reinforcement as a crucial process for memory consolidation. Science. 2000;290(5494):1170–1174.

[4] Szapiro G, Vianna MRM, McGaugh JL, Medina JH, Izquierdo I. The role of NMDA glutamate receptors, PKA, MAPK, and CAMKII in the hippocampus in extinction of conditioned fear. Hippocampus. 2003;13(1):53–58. doi:10.1002/hipo.10043.

[5] O'Dell TJ, Connor SA, Guglietta R, Nguyen PV. β-Adrenergic receptor signaling and modulation of long-term potentiation in the mammalian hippocampus. Learn Mem. 2015;22:461–467. doi:10.1101/lm.031088.113.

[6] Wilson CB, Ebenezer PJ, McLaughlin LD, Francis J. Predator Exposure/Psychosocial Stress Animal Model of Post-Traumatic Stress Disorder Modulates Neurotransmitters in the Rat Hippocampus and Prefrontal Cortex. PLoS One. 2014;9(2):e89104. doi:10.1371/journal.pone.0089104.

[7] Kao CY, Stalla G, Stalla J, Wotjak CT, Anderzhanova E. Norepinephrine and corticosterone in the medial prefrontal cortex and hippocampus predict PTSD-like symptoms in mice. Eur J Neurosci. 2015;41(9):1139–1148. doi:10.1111/ejn.12860.

[8] Giese KP, Mizuno K. The roles of protein kinases in learning and memory. Learn Mem. 2013;20:540–52. doi:10.1101/lm.028449.112.

[9] Woo NH, Duffy SN, Abel T, Nguyen PV. Temporal spacing of synaptic stimulation critically modulates the dependence of LTP on cyclic AMP-dependent protein kinase. Hippocampus. 2003;13(2):293–300. doi:10.1002/hipo.10086.

[10] Kim M, Huang T, Abel T, Blackwell KT. Temporal sensitivity of protein kinase A activation in late-phase long term potentiation. PLoS Comput Biol. 2010;6(2):e1000691. doi:10.1371/journal.pcbi.1000691.

[11] English JD, Sweatt JD. A requirement for the mitogen-activated protein kinase cascade in hippocampal long term potentiation. J Biol Chem. 1997;272(31):19103–19106. doi:10.1074/jbc.272.31.19103.

[12] Winder DG, Martin KC, Muzzio IA, Rohrer D, Chruscinski A, Kobilka B, et al. ERK plays a regulatory role in induction of LTP by theta frequency stimulation and its modulation by β-adrenergic receptors. Neuron. 1999;24(3):715–26.

[13] Thomas GM, Huganir RL. MAPK cascade signalling and synaptic plasticity. Nat Rev Neurosci. 2004;5(3):173–83. doi:10.1038/nrn1346.

[14] Gelinas JN, Banko JL, Peters MM, Klann E, Weeber EJ, Nguyen PV. Activation of exchange protein activated by cyclic-AMP enhances long-lasting synaptic potentiation in the hippocampus. Learn Mem. 2008;15(6):403–11. doi:10.1101/lm.830008.

[15] Luttrell LM, Hawes BE, van Biesen T, Luttrell DK, Lansing TJ, Lefkowitz RJ. Role of c-Src Tyrosine Kinase in G protein-coupled receptor and $G_{\beta\gamma}$ subunit-mediated activation of mitogen activated protein kinases. J Biol Chem. 1996;271(32):19443–19450. doi:10.1074/jbc.271.32.19443.

[16] Della Rocca GJ, van Biesen T, Daaka Y, Luttrell DK, Luttrell LM, Lefkowitz RJ. Ras-dependent mitogen-activated protein kinase activation by G protein-coupled receptors: convergence of G_i- and G_q-mediated pathways on calcium/calmodulin, Pyk2, and Src kinase. J Biol Chem. 1997;272(31):19125–19132. doi:10.1074/jbc.272.31.19125.

[17] Martin NP, Whalen EJ, Zamah Ma, Pierce KL, Lefkowitz RJ. PKA-mediated phosphorylation of the β1-adrenergic receptor promotes G_s/G_i switching. Cell Signal. 2004;16(12):1397–403. doi:10.1016/j.cellsig.2004.05.002.

[18] Kahsai AW, Xiao K, Rajagopal S, Ahn S, Shukla AK, Sun J, et al. Multiple ligand-specific conformations of the β2-adrenergic receptor. Nat Chem Biol. 2011;7(10):692–700. doi:10.1038/nchembio.634.

[19] Havekes R, Canton DA, Park AJ, Huang T, Nie T, Day JP, et al. Gravin orchestrates protein kinase A and β2-adrenergic receptor signaling critical for synaptic plasticity and memory. J Neurosci. 2012;32(50):18137–18149. doi:10.1523/JNEUROSCI.3612-12.2012.

[20] Kim M, Park AJ, Havekes R, Chay A, Guercio LA, Oliveira RF, et al. Colocalization of protein kinase A with adenylyl cyclase enhances protein kinase A activity during induction of long-lasting long-term-potentiation. PLoS Comput Biol. 2011;7(6):e1002084. doi:10.1371/journal.pcbi.1002084.

[21] Kubota Y, Putkey JA, Waxham MN. Neurogranin controls the spatiotemporal pattern of postsynaptic Ca^{2+}/CaM signaling. Biophys J. 2007;93(11):3848–59. doi:10.1529/biophysj.107.106849.

[22] Zhabotinsky AM, Camp RN, Epstein IR, Lisman JE. Role of the neurogranin concentrated in spines in the induction of long-term potentiation. J Neurosci. 2006;26(28):7337–47. doi:10.1523/JNEUROSCI.0729-06.2006.

[23] Zhong L, Brown J, Kramer A, Kaleka K, Petersen A, Krueger JN, et al. Increased prefrontal cortex neurogranin enhances plasticity and extinction learning. J Neurosci. 2015;35(19):7503–7508. doi:10.1523/JNEUROSCI.0274-15.2015.

[24] Daaka Y, Luttrell LM, Lefkowitz RJ. Switching of the coupling of the β2-adrenergic receptor to different G proteins by protein kinase A. Nature. 1997;390(6655):88–91. doi:10.1038/36362.

[25] Tang WJ, Krupinski J, Gilman AG. Expression and characterization of calmodulin-activated (type I) adenylyl cyclase. J Biol Chem. 1991;266(13):8595–8603.

[26] Hillman KL, Doze Va, Porter JE. Functional characterization of the beta-adrenergic receptor subtypes expressed by CA1 pyramidal cells in the rat hippocampus. Pharmacology. 2005;314(2):561–567. doi:10.1124/jpet.105.084947.).

[27] Sibley D, Lefkowitz RJ. Molecular mechanisms of receptor desensitization using the β-adrenergic receptor-coupled adenylate cyclase system as a model. Nature. 1985;317:124. doi:10.1038/317124a0.

[28] Liggett SB, Bouvier M, Hausdorff WP, O'Dowd B, Caron MG, Lefkowitz RJ. Altered patterns of agonist-stimulated cAMP accumulation in cells expressing mutant beta 2-adrenergic receptors lacking phosphorylation sites. Mol Pharmacol. 1989;36(4):641–646.

[29] Chay A, Zamparo I, Koschinski A, Zaccolo M, Blackwell KT. Control of β2AR- and N-methyl-D-aspartate (NMDA) receptor-dependent cAMP dynamics in hippocampal neurons. PLoS Comput Biol. 2015;12(2). doi:10.1371/journal.pcbi.1004735.

[30] Thomson M, Gunawardena J. The rational parameterization theorem for multisite posttranslational modification systems. J Theor Biol. 2009;261:626. doi:10.1016/j.jtbi.2009.09.003.

[31] Ferrell JE Jr, Bhatt RR. Mechanistic studies of the dual phosphorylation of mitogen-activated protein kinase. J Biol Chem. 1997;272:19008. doi:10.1074/jbc.272.30.19008.

[32] Dushek O, van der Merwe PA, Shahrezaei V. Ultrasensitivity in multisite phosphorylation of membrane-anchored proteins. Biophys J. 2011;100:1189.

[33] Shenoy SK, Drake MT, Nelson CD, Houtz DA, Xiao K, Madabushi S, et al. β-arrestin-dependent, G protein-independent ERK1/2 activation by the β2-adrenergic receptor. J Biol Chem. 2006;281:1261–1273. doi:10.1074/jbc.M506576200.

[34] Bacskai BJ, Hochner B, Mahaut-Smith M, Adams SR, Kaang BK, Kandel ER, Tsien RY Spatially resolved dynamics of cAMP and protein kinase A subunits in Aplysia sensory neurons. Science. 1993;260:222–226.

[35] Mironov SL, Skorova E, Taschenberger G, Hartelt N, Nikolaev VO, Lohse MJ, Kugler S Imaging cytoplasmic cAMP in mouse brainstem neurons. BMC Neurosc. 2009;10:29. doi:10.1186/1471-2202-10-2

[36] Antunes G, De Shcutter E. A stochastic signaling network mediates the probabilistic induction of cerebellar long-term depression. J Neurosci. 2012;32(27):9288–9300. doi:10.1523/JNEUROSCI.5976-11.2012.

[37] Gillespie DT. Exact stochastic simulation of coupled chemical reactions. J Phys Chem. 1977;93555(1):2340–2361.

[38] Oliveira RF, Terrin A, Di Benedetto G, Cannon RC, Koh W, Kim M, et al. The role of type 4 phosphodiesterases in generating microdomains of cAMP: large scale stochastic simulations. PLoS One. 2010;5(7):e11725. doi:10.1371/journal.pone.0011725.

[39] Lisman JE, Raghavachari S, Otmakhov N, Otmakhova NA. The Phases of LTP: The New Complexities. In: Stanton PK, Bramham C, Scharfman HE, editors. Synaptic Plasticity and Transsynaptic Signaling. Springer US; 2005. p. 343–357. Available from: http://dx.doi.org/10.1007/0-387-25443-9_20.

[40] Milusheva E, Baranyi M, Zelles T, Mike A, Vizi ES. Release of acetylocholine and noradrenaline from the cholinergic and adrenergic afferents in rat hippocampal CA1, CA3 and dentate gyrus regions. Eur J Neurosci. 1994;6:187. doi:10.1111/j.1460-9568.1994.tb00260.x.

[41] Kim E, Owen B, Holmes WR, Grover LM. Decreased afferent excitability contributes to synaptic depression during high-frequency stimulation in hippocampal area CA1. J Neurophysiol. 2012;108(7):1965–76. doi:10.1152/jn.00276.2011.

[42] Scheuss V, Yasuda R, Sobczyk A, Svoboda K. Nonlinear Ca^{2+} signaling in dendrites and spines caused by activity-dependent depression of Ca^{2+} extrusion. J Neurosci. 2006;26(31):8183–94. doi:10.1523/JNEUROSCI.1962-06.2006.

[43] Tsodyks MV, Pawelzik K, Markram H. Neural Networks with Dynamic Synapses. Neural Comput. 1998;10(1):821. doi:10.1162/089976698300017502.

[44] Park J, Kile BM, Wightman RM. In vivo voltammetric monitoring of norepinephrine release in the rat ventral bed nucleus of the stria terminalis and anteroventral thalamic nucleus. Eur J Neurosci. 2009;30(11):2121–2133. doi:10.1111/j.1460-9568.2009.07005.x.In.

[45] Dreyer JK, Herrik KF, Berg RW, Hounsgaard JrD. Influence of phasic and tonic dopamine release on receptor activation. J Neurosci. 2010;30(42):14273–83. doi:10.1523/JNEUROSCI.1894-10.2010.

[46] Skeberdis VA, Chavaleyre V, Lau CG, Goldberg JH, Petti DL, Suadicani SO, et al. Protein kinase A regulates calcium permeability of NMDA receptors. Nat Neurosci. 2006;9(4):501. doi:10.1038/nn1664.

[47] Murphy JA, Stein IS, Lau CG, Peixoto RT, Aman TK, Kaneko N, et al. Phosphorylation of Ser1166 on GluN2B by PKA is critical to synaptic NMDA receptor function and Ca^{2+} signaling in spines. J Neurosci. 2014;34(3):869–79. doi:10.1523/JNEUROSCI.4538-13.2014.

[48] Liebmann L, Karst H, Joëls M. Effects of corticosterone and the β-agonist isoproterenol on glutamate receptor-mediated synaptic currents in the rat basolateral amygdala. Eur J Neurosci. 2009;30(5):800–807. doi:10.1111/j.1460-9568.2009.06882.x.

[49] Swanson-Park JL, Coussens CM, Mason-Parker SE, Raymond CR, Hargreaves EL, Dragunow M, et al. A double dissociation within the hippocampus of dopamine D1/D5 receptor and β-adrenergic receptor contributions to the persistence of long-term potentiation. Neuroscience. 1999;92(2):485–497. doi:10.1016/S0306-4522(99)00010-X.

[50] Tzingounis AV, von Zastrow M, Yudowski Ga. β-blocker drugs mediate calcium signaling in native central nervous system neurons by β-arrestin-biased agonism. Proc Natl Acad Sci U S A. 2010;107(49):21028–33. doi:10.1073/pnas.1004169107.

[51] Smith C, Teitler M. β-blocker selectivity at cloned human β1- and β2-adrenergic receptors. Cardiovasc Drugs Ther. 1999;13(2):123–126. doi:10.1023/A:1007784109255.

[52] Gelinas JN, Nguyen PV. β-adrenergic receptor activation facilitates induction of a protein synthesis-dependent late phase of long-term potentiation. J Neurosci. 2005;25(13):3294–303. doi:10.1523/JNEUROSCI.4175-04.2005.

[53] Duffy SN, Craddock KJ, Abel T, Nguyen PV. Environmental enrichment modifies the PKA-dependence of hippocampal LTP and improves hippocampus-dependent memory. Learn Mem. 2001;8(1):26–34. doi:10.1101/lm.36301.

[54] Huang YY, Kandel ER. Recruitment of long-lasting and protein kinase A-dependent long-term potentiation in the CA1 region of hippocampus requires repeated tetanization. Learn Mem. 1994;1:74–82. doi:10.1101/lm.1.1.74.

[55] Sanhueza M, Fernandez-Villalobos G, Stein IS, Kasumova G, Zhang P, Bayer KU, et al. Role of the CaMKII/NMDA receptor complex in the maintenance of synaptic strength. J Neurosci. 2011;31(25):9170–8. doi:10.1523/JNEUROSCI.1250-11.2011.

[56] Zhang M, Storm DR, Wang H. Bidirectional synaptic plasticity and spatial memory flexibility require Ca^{21+}-stimulated adenylyl cyclases. J Neurosci. 2011;31(28):10174–83. doi:10.1523/JNEUROSCI.0009-11.2011.

[57] Abel T, Nguyen PV, Barad M, Deuel TAS, Kandel ER, Bourtchouladze R. Genetic Demonstration of a Role for PKA in the Late Phase of LTP and in Hippocampus-Based Long-Term Memory. Cell. 1997;88(5):615–626. doi:10.1016/S0092-8674(00)81904-2.

[58] Redondo RL, Okuno H, Spooner Pa, Frenguelli BG, Bito H, Morris RGM. Synaptic tagging and capture: differential role of distinct calcium/calmodulin kinases in protein synthesis-dependent long-term potentiation. J Neurosci. 2010;30(14):4981–9. doi:10.1523/JNEUROSCI.3140-09.2010.

[59] Sajikumar S, Navakkode S, Frey JU. Identification of compartment- and process-specific molecules required for "synaptic tagging" during long-term potentiation and long-term depression in hippocampal CA1. J Neurosci. 2007;27(19):5068–80. doi:10.1523/JNEUROSCI.4940-06.2007.

[60] Ramachandran B, Frey JU. Interfering with the actin network and its effect on long-term potentiation and synaptic tagging in hippocampal CA1 neurons in slices in vitro. J Neurosci. 2009;29(39):12167–73. doi:10.1523/JNEUROSCI.2045-09.2009.

[61] Murakoshi H, Shin ME, Parra-Bueno P, Szatmari E, Shibata ACE, Yasuda R. Kinetics of Endogenous CaMKII Required for Synaptic Plasticity Revealed by Optogenetic Kinase Inhibitor. Neuron. 2017;94(1):37–47. doi:10.1016/j.neuron.2017.02.036.

[62] Frey U, Morris R. Synaptic tagging and long-term potentiation. Nature. 1997;385:533—-536.

[63] Young JZ, Isiegas C, Abel T, Nguyen PV. Metaplasticity of the late-phase of long-term potentiation: a critical role for protein kinase A in synaptic tagging. Eur J Neurosci. 2006;23(7):1784–94. doi:10.1111/j.1460-9568.2006.04707.x.

[64] Luczak V, Blackwell KT, Abel T, Girault JA, Gervasi N. Dendritic diameter influences the rate and magnitude of hippocampal cAMP and PKA transients during β-adrenergic receptor activation. Neurobiol Learn Mem. 2017;138:10–20. doi:10.1016/j.nlm.2016.08.006.

[65] Vanhoose AM, Winder DG. NMDA and β1-Adrenergic Receptors Differentially Signal Phosphorylation of Glutamate Receptor Type 1 in Area CA1 of Hippocampus. J Neurosci. 2004;23 (13) 5827-5834.

[66] Gray EE, Guglietta R, Khakh BS, O'Dell TJ Inhibitory Interactions between Phosphorylation Sites in the C-terminus of α-Amino-3-Hydroxy-5-Methyl-4-Isoxazolepropionic Acid-Type Glutamate Receptor GluA1 Subunits. J Biol Chem. 2014;289(21):14600-11. doi:10.1074/jbc.M114.553537.

[67] Brown G, Blitzer R, Connor J, Wong T, Shenolikar S, Iyengar R, et al. Long-term potentiation induced by theta frequency stimulation is regulated by a protein phosphatase-1-operated gate. jn. 2000;20:7887.

[68] MacKenzie SJ, Baillie GS, McPhee I, MacKenzie C, Seamons R, McSorley T, et al. Long PDE4 cAMP specific phosphodiesterases are activated by protein kinase A-mediated phosphorylation of a single serine residue in Upstream Conserved Region 1 (UCR1). Br J Pharmacol. 2002;136(3):421–33. doi:10.1038/sj.bjp.0704743.

[69] Blitzer R, Connor J, Brown G, Wong T, Shenolikar S, Iyengar R, et al. Gating of CaMKII by cAMP-regulated protein phosphatase activity during LTP. Science. 1998;280:1940. doi:10.1126/science.280.5371.1940.

[70] Lee HK, Barbarosie M, Kameyama K, Bear MF, Huganir RL. Regulation of distinct AMPA receptor phosphorylation sites during bidirectional synaptic plasticity. Nature. 2000;405(6789):955–9. doi:10.1038/35016089.

[71] Qian H, Matt L, Zhang M, Nguyen M, Patriarchi T, Koval OM, et al. β2-Adrenergic receptor supports prolonged theta tetanus-induced LTP. J Neurophysiol. 2012;107(10):2703–12. doi:10.1152/jn.00374.2011.

[72] Matsuzaki M, Honkura N, Ellis-Davies G, Kasai H. Structural basis of long-term potentiation in single dendritic spines. Nature. 2004;479(June):761–766. doi:10.1038/nature02594.1.

[73] Harvey CD, Yasuda R, Zhong H, Svoboda K. The apread of Ras activity triggered by activation of a single dendritic spine. Science. 2008;321(5885):136–140. doi:10.1126/science.1159675.

[74] Govindarajan A, Israely I, Huang SY, Tonegawa S. The dendritic branch is the preferred integrative unit for protein synthesis-dependent LTP. Neuron. 2011;69(1):132–46. doi:10.1016/j.neuron.2010.12.008.

[75] Zhai S, Ark ED, Parra-Bueno P, Yasuda R. Long-distance integration of nuclear ERK signaling triggered by activation of a few dendritic spines. Science. 2013;342(6162):1107–1111. doi:10.1126/science.1245622.

[76] Lee SJR, Escobedo-Lozoya Y, Szatmari EM, Yasuda R. Activation of CaMKII in single dendritic spines during long-term potentiation. Nature. 2009;458(7236):299–304. doi:10.1038/nature07842.

[77] Huber KM, Mauk MD, Thompson C, Kelly PT. A critical period of protein kinase activity after tetanic stimulation is required for the induction of long-term potentiation. Learning & Memory. 1995;2:81–100. doi:10.1101/lm.2.2.81.

[78] Redondo RL, Morris RGM. Making memories last: the synaptic tagging and capture hypothesis. Nat Rev Neurosci. 2011;12(1):17–30. doi:10.1038/nrn2963.

[79] Okamoto KI, Nagai T, Miyawaki A, Hayashi Y. Rapid and persistent modulation of actin dynamics regulates postsynaptic reorganization underlying bidirectional plasticity. Nat Neurosci. 2004;7(10):1104–12. doi:10.1038/nn1311.

[80] Nishiyama J, Yasuda R. Biochemical Computation for Spine Structural Plasticity. Neuron. 2015;87(1):63 – 75. doi:http://dx.doi.org/10.1016/j.neuron.2015.05.043.

[81] Patterson MA, Szatmari EM, Yasuda R. AMPA receptors are exocytosed in stimulated spines and adjacent dendrites in a Ras-ERK–dependent manner during long-term potentiation. Proc Natl Acad Sci U S A. 2010;107(36):15951–6. doi:10.1073/pnas.0913875107.

[82] Yang Y, Wang Xb, Frerking M, Zhou Q. Spine Expansion and Stabilization Associated with Long-Term Potentiation. J Neurosci. 2008;28(22):5740–5751. doi:10.1523/JNEUROSCI.3998-07.2008.

[83] Shen K, Teruel MN, Subramanian K, Meyer T. CaMKIIβ functions as an F-actin targeting module that localizes CaMKIIα/β heterooligomers to dendritic spines. Neuron. 1998;21(3):593. doi:10.1016/S0896-6273(00)80569-3.

[84] Khan S, Zou Y, Amjad A, Gardezi A, Smith CL, Winters C, et al. Sequestration of CaMKII in dendritic spines in silico. J Comput Neurosci. 2011;31(3):581–94. doi:10.1007/s10827-011-0323-2.

[85] Khan S, Reese TS, Rajpoot N, Shabbir A. Spatiotemporal maps of CaMKII in dendritic spines. J Comput Neurosci. 2012;33(1):123–39. doi:10.1007/s10827-011-0377-1.

[86] Araki Y, Zeng M, Zhang M, Huganir RL. Rapid dispersion of SynGAP from synaptic spines triggers AMPA receptor insertion and spine enlargement during LTP. Neuron. 2015;85(1):173–189. doi:10.1016/j.neuron.2014.12.023.

[87] Navakkode S, Sajikumar S, Frey JU. The type IV-specific phosphodiesterase inhibitor rolipram and its effect on hippocampal long-term potentiation and synaptic tagging. J Neurosci. 2004;24(35):7740–4. doi:10.1523/JNEUROSCI.1796-04.2004.

[88] Huang T, McDonough CB, Abel T. Compartmentalized PKA signaling events are required for synaptic tagging and capture during hippocampal late-phase long-term potentiation. Eur J Cell Biol. 2006;85(7):635. doi:10.1016/j.ejcb.2006.02.005.

[89] Lamprecht R, LeDoux J. Structural plasticity and memory. Nat Rev Neurosci. 2004;5(1):45–54. doi:10.1038/nrn1301.

[90] Nadella KS, Saji M, Jacob NK, Pavel E, Ringel MD, Kirschner LS. Regulation of actin function by protein kinase A-mediated phosphorylation of Limk1. EMBO Rep. 2009;10(6):599–605. doi:10.1038/embor.2009.58.

[91] Chen LY, Rex CS, Casale MS, Gall CM, Lynch G. Changes in synaptic morphology accompany actin signaling during LTP. J Neurosci. 2007;27(20):5363–5372. doi:10.1523/JNEUROSCI.0164-07.2007.

[92] Gu J, Lee CW, Fan Y, Komlos D, Tang X, Sun C, et al. ADF/cofilin-mediated actin dynamics regulate AMPA receptor trafficking during synaptic plasticity. Nat Neurosci. 2010;13(10):1208–1215.

[93] Penzes P, Woolfrey KM, Srivastava DP. Epac2-mediated dendritic spine remodeling: implications for disease. Mol Cell Neurosci. 2011;46(2):368–80. doi:10.1016/j.mcn.2010.11.008.

[94] Penzes P, Cahill ME. Deconstructing signal transduction pathways that regulate the actin cytoskeleton in dendritic spines. Cytoskeleton. 2012;69(7):426. doi:10.1002/cm.21015.

[95] Vossler MR, Yao H, York RD, Pan MG, Rim CS, Stork PJS. cAMP activates MAP kinase and Elk-1 through a B-Raf- and Rap1-dependent pathway. Cell. 1997;89(1):73–82. doi:10.1016/S0092-8674(00)80184-1.

[96] Lin SL, Johnson-Farley NN, Lubinsky DR, Cowen DS. Coupling of neuronal 5-HT7 receptors to activation of extracellular-regulated kinase through a protein kinase A-independent pathway that can utilize Epac. J Neurochem. 2003;87(5):1076–1085. doi:10.1046/j.1471-4159.2003.02076.x.

[97] Wang Z, Dillon TJ, Pokala V, Mishra S, Labudda K, Hunter B, et al. Rap1-mediated activation of extracellular signal-regulated kinases by cyclic AMP is dependent on the mode of Rap1 activation. Mol Cell Biol. 2006;26(6):2130–2145. doi:10.1128/MCB.26.6.2130.

[98] McAvoy T, Zhou Mm, Greengard P, Nairn AC. Phosphorylation of Rap1GAP, a striatally enriched protein, by protein kinase A controls Rap1 activity and dendritic spine morphology. Proc Natl Acad Sci U S A. 2009;106(9):3531–6. doi:10.1073/pnas.0813263106.

[99] Kelleher RJ, Govindarajan A, Tonegawa S. Translational regulatory mechanisms in persistent forms of synaptic plasticity. Neuron. 2004;44(1):59–73. doi:10.1016/j.neuron.2004.09.013.

[100] Gelinas JN, Banko JL, Hou L, Sonenberg N, Weeber EJ, Klann E, et al. ERK and mTOR signaling couple β-adrenergic receptors to translation initiation machinery to gate induction of protein synthesis-dependent long-term potentiation. J Biol Chem. 2007;282(37):27527–35. doi:10.1074/jbc.M701077200.

[101] Ajay SM, Bhalla US. A propagating ERKII switch forms zones of elevated dendritic activation correlated with plasticity. HFSP J. 2007;1(1):49. doi:10.2976/1.2721383.

[102] Atkins CM, Nozaki N, Shigeri Y, Soderling TR. Cytoplasmic polyadenylation element binding protein-dependent protein synthesis is regulated by Calcium/Calmodulin-dependent protein kinase II. J Neurosci. 2004;24(22):5193. doi:10.1523/JNEUROSCI.0854-04.2004.

[103] Atkins CM, Davare MA, Oh MC, Derkach V, Soderling TR. Bidirectional Regulation of Cytoplasmic Polyadenylation Element-Binding Protein Phosphorylation by Ca2+/Calmodulin-Dependent Protein Kinase II and Protein Phosphatase 1 during Hippocampal Long-Term Potentiation. J Neurosci. 2005;25(23):5604. doi:10.1523/JNEUROSCI.5051-04.2005.

[104] Jain P, Bhalla US. Transcription control pathways decode patterned synaptic inputs into diverse mRNA expression profiles. PLoS One. 2014;9(5):e95154. doi:10.1371/journal.pone.0095154.

[105] Clopath C, Ziegler L, Vasilaki E, Büsing L, Gerstner W. Tag-Trigger-Consolidation: A Model of Early and Late Long-Term-Potentiation and Depression. PLoS Comput Biol. 2008;4(12):e1000248. doi:10.1371/journal.pcbi.1000248.

[106] Barrett AB, Billings GO, Morris RGM, van Rossum MCW. State based model of long-term potentiation and synaptic tagging and capture. PLoS Comput Biol. 2009;5(1):e1000259. doi:10.1371/journal.pcbi.1000259.

[107] Smolen P, Baxter DA, Byrne JH. Molecular constraints on synaptic tagging and maintenance of long-term potentiation: a predictive model. PLoS Comput Biol. 2012;8(8):e1002620. doi:10.1371/journal.pcbi.1002620.

[108] Smolen P, Baxter DA, Byrne JH. Simulations suggest pharmacological methods for rescuing long-term potentiation. J Theor Biol. 2014;360:243. doi:10.1016/j.jtbi.2014.07.006.

[109] Kuo MH, Allis CD. Roles of histone acetyltransferases and deacetylases in gene regulation. BioEssays. 1998;20(8):615–626. doi:10.1002/(SICI)1521-1878(199808)20:8<615::AID-BIES4>3.0.CO;2-H.

[110] Lee HK, Takamiya K, Han JS, Man H, Kim CH, Rumbaugh G, et al. Phosphorylation of the AMPA receptor GluR1 subunit is required for synaptic plasticity and retention of spatial memory. Cell. 2003;112(5):631–43. doi:10.1016/S0092-8674(03)00122-3.

[111] Lee HK, Takamiya K, He K, Song L, Huganir RL. Specific roles of AMPA receptor subunit GluR1 (GluA1) phosphorylation sites in regulating synaptic plasticity in the CA1 region of hippocampus. J Neurophysiol. 2010;103(1):479–89. doi:10.1152/jn.00835.2009.

[112] Esteban JA, Shi SH, Wilson C, Nuriya M, Huganir RL, Malinow R. PKA phosphorylation of AMPA receptor subunits controls synaptic trafficking underlying plasticity. Nat Neurosci. 2003;6(2):136–43. doi:10.1038/nn997.

[113] Hayer A, Bhalla US. Molecular switches at the synapse emerge from receptor and kinase traffic. PLoS Comput Biol. 2005;1(2):137–54. doi:10.1371/journal.pcbi.0010020.

[114] Huang YY, Kandel ER. D1/D5 receptor agonists induce a protein synthesis-dependent late potentiation in the CA1 region of the hippocampus. Proc Natl Acad Sci U S A. 1995;92(7):2446–2450. doi:10.1073/pnas.92.7.2446.

[115] Sedova M, Blatter La. Dynamic regulation of Ca^{2+}i by plasma membrane Ca^{2+}-ATPase and Na+/Ca^{2+} exchange during capacitative Ca^{2+} entry in bovine vascular endothelial cells. Cell Calcium. 1999;25:333–343. doi:10.1054/ceca.1999.0036.

[116] Lörincz A, Rózsa B, Katona G, Vizi ES, Tamás G. Differential distribution of NCX1 contributes to spine-dendrite compartmentalization in CA1 pyramidal cells. Proc Natl Acad Sci U S A. 2007;104(3):1033–1038. doi:10.1073/pnas.0605412104.

[117] Gall D, Gromada J, Susa I, Rorsman P, Herchuelz A, Bokvist K. Significance of Na/Ca exchange for Ca^{2+} buffering and electrical activity in mouse pancreatic β-cells. Biophys J. 1999;76(4):2018–2028. doi:10.1016/S0006-3495(99)77359-5.

[118] Schmidt H, Kunerth S, Wilms C, Strotmann R, Eilers J. Spino-dendritic cross-talk in rodent Purkinje neurons mediated by endogenous Ca^{2+}-binding proteins. J Physiol. 2007;581(Pt 2):619–629. doi:10.1113/jphysiol.2007.127860.

[119] Brown SE, Martin SR, Bayley PM. Kinetic control of the dissociation pathway of calmodulin-peptide complexes. J Biol Chem. 1997;272(6):3389–3397. doi:10.1074/jbc.272.6.3389.

[120] Putkey JA, Kleerekoper Q, Gaertner TR, Waxham MN. A new role for IQ motif proteins in regulating calmodulin function. J Biol Chem. 2003;278(50):49667–70. doi:10.1074/jbc.C300372200.

[121] Quintana AR, Wang D, Forbes JE, Waxham MN. Kinetics of calmodulin binding to calcineurin. Biochem Biophys Res Commun. 2005;334(2):674–80. doi:10.1016/j.bbrc.2005.06.152.

[122] Stemmer PM, Klee CB. Dual calcium ion regulation of calcineurin by calmodulin and calcineurin B. Biochemistry. 1994;33(22):6859–6866. doi:10.1021/bi00188a015.

[123] Gaertner TR, Putkey JA, Waxham MN. RC3/Neurogranin and Ca^{2+}/calmodulin-dependent protein kinase II produce opposing effects on the affinity of calmodulin for calcium. J Biol Chem. 2004;279(38):39374–82. doi:10.1074/jbc.M405352200.

[124] Dupont G, Goldbeter A. CaM kinase II as frequency decoder of Ca^{2+} oscillations. BioEssays. 1998;20(8):607–610. doi:10.1002/(SICI)1521-1878(199808)20:8<607::AID-BIES2>3.0.CO;2-F.

[125] Foulkes JG, Strada SJ, Henderson PJ, Cohen P. A kinetic analysis of the effects of inhibitor-1 and inhibitor-2 on the activity of protein phosphatase-1. FEBS J. 1983;132(2):309–13.

[126] Bradshaw JM, Kubota Y, Meyer T, Schulman H. An ultrasensitive Ca^{2+}/calmodulin-dependent protein kinase II-protein phosphatase 1 switch facilitates specificity in postsynaptic calcium signaling. Proc Natl Acad Sci U S A. 2003;100(18):10512–7. doi:10.1073/pnas.1932759100.

[127] Garnier V, Zini R, Sapena R, Tillement JP. A match between binding to β-adrenoceptors and stimulation of adenylyl cyclase parameters of (-)isoproterenol and salbutamol on rat brain. Pharmacol Res. 1997;35(4):303–312. doi:10.1006/phrs.1997.0141.

[128] Falkenburger BH, Jensen JB, Hille B. Kinetics of M1 muscarinic receptor and G protein signaling to phospholipase C in living cells. The Journal of general physiology. 2010;135(2):81–97. doi:10.1085/jgp.200910345.

[129] Liu HY, Wenzel-Seifert K, Seifert R. The olfactory G protein $G_{\alpha olf}$ possesses a lower GDP-affinity and deactivates more rapidly than $G_{s\alpha short}$: consequences for receptor-coupling and adenylyl cyclase activation. J Neurochem. 2001;551:325–338. doi:10.1046/j.1471-4159.2001.00422.x.

[130] Mukhopadhyay S, Ross EM. Rapid GTP binding and hydrolysis by G_q promoted by receptor and GTPase-activating proteins. Proc Natl Acad Sci U S A. 1999;96(August):9539–9544. doi:10.1073/pnas.96.17.9539.

[131] Berman DM, Wilkie TM, Gilman AG. GAIP and RGS4 are GTPase-activating proteins for the G_i subfamily of G protein α subunits. Cell. 1996;86:445–452. doi:10.1016/S0092-8674(00)80117-8.

[132] Neubig RR, Connolly MP, Remmers aE. Rapid kinetics of G protein subunit association: a rate-limiting conformational change? FEBS Let. 1994;355:251–253. doi:10.1016/0014-5793(94)01212-1.

[133] Sykes D, Parry C, Reilly J, Wright P, Fairhurst RA, Charlton S. Observed drug-receptor association rates are governed by membrane affinity: the importance of establishing "micro-pharmacokinetic/pharmacodynamic relationships" at the β2-adrenoceptor. Mol Pharmacol. 2014;85(4):608–17. doi:10.1124/mol.113.090209.

[134] Tran TM, Friedman J, Baameur F, Knoll BJ, Moore RH, Clark RB. Characterization of β2-adrenergic receptor dephosphorylation: Comparison with the rate of resensitization. Mol Pharmacol. 2007;71(1):47–60. doi:10.1124/mol.106.028456.

[135] Harrison JK, Keikilani Hewlett GH, Gnegy ME. Regulation of calmodulin-sensitive adenylate cyclase by the stimulatory G-protein, G_s. J Biol Chem. 1989;264(27):15880–15885.

[136] Wang H, Storm DR. Calmodulin-regulated adenylyl cyclases: cross-talk and plasticity in the central nervous system. Mol Pharmacol. 2003;63(3):463–468. doi:10.1124/mol.63.3.463.

[137] Wayman GA, Impey S, Wu Z, Kindsvogel W, Prichard L, Storm DR. Synergistic activation of the type I adenylyl cyclase by Ca^{2+} and G_s-coupled receptors in vivo. J Biol Chem. 1994;269(41):25400–25405.

[138] Chen-Goodspeed M, Lukan AN, Dessauer CW. Modeling of $G_{\alpha s}$ and $G_{\alpha i}$ regulation of human type V and VI adenylyl cyclase. J Biol Chem. 2005;280(3):1808–1816. doi:10.1074/jbc.M409172200.

[139] Nielsen MD, Chan GCK, Poser SW, Storm DR. Differential regulation of type I and type VIII Ca^{2+}-stimulated adenylyl cyclases by G_i-coupled receptors in vivo. J Biol Chem. 1996;271(52):33308–33316. doi:10.1074/jbc.271.52.33308.

[140] Cali JJ, Parekh RS, Krupinski J. Splice variants of type VIII adenylyl cyclase. Differences in glycosylation and regulation by Ca^{2+}/calmodulin. J Biol Chem. 1996;271(2):1089–1095. doi:10.1074/jbc.271.2.1089.

[141] Nikolaev VO, Bünemann M, Hein L, Hannawacker A, Lohse MJ. Novel single chain cAMP sensors for receptor-induced signal propagation. J Biol Chem. 2004;279(36):37215–8. doi:10.1074/jbc.C400302200.

[142] Ogreid D, Døskeland SO. The kinetics of association of cyclic AMP to the two types of binding sites associated with protein kinase II from bovine myocardium. FEBS Let. 1981;129(2):287–292.

[143] Herberg FW, Taylor SS, Dostmann WR. Active site mutations define the pathway for the cooperative activation of cAMP-dependent protein kinase. Biochemistry. 1996;35(9):2934–42. doi:10.1021/bi951647c.

[144] Ogreid D, Døskeland SO. Activation of protein kinase isoenzymes under near physiological conditions. Evidence that both types (A and B) of cAMP binding sites are involved in the activation of protein kinase by cAMP and 8-N3-cAMP. FEBS Let. 1982;150(1):161–6.

[145] Zawadzki KM, Taylor SS. cAMP-dependent protein kinase regulatory subunit type IIβ: active site mutations define an isoform-specific network for allosteric signaling by cAMP. J Biol Chem. 2004;279(8):7029–36. doi:10.1074/jbc.M310804200.

[146] Hemmings HC, Nairn AC, Greengard P. DARPP-32, a dopamine- and adenosine-3':5'-monophosphate-regulated neuronal phosphoprotein. II. Comparison of the kinetics of phosphorylation of DARPP-32 and phosphatase inhibitor 1. J Biol Chem. 1984;259(23):14491–14497.

[147] Huang HB, Horiuchi A, Watanabe T, Shih SR, Tsay HJ, Li HC, et al. Characterization of the inhibition of protein phosphatase-1 by DARPP-32 and inhibitor-2. J Biol Chem. 1999;274(12):7870–7878. doi:10.1074/jbc.274.12.7870.

[148] Connor JH, Frederick D, Huang HB, Yang J, Helps NR, Cohen PT, et al. Cellular mechanisms regulating protein phosphatase-1. A key functional interaction between inhibitor-2 and the type 1 protein phosphatase catalytic subunit. J Biol Chem. 2000;275(25):18670–18675. doi:10.1074/jbc.M909312199.

[149] Desdouits F, Siciliano JC, Greengard P, Girault JA. Dopamine- and cAMP-regulated phosphoprotein DARPP-32: phosphorylation of Ser-137 by casein kinase I inhibits dephosphorylation of Thr-34 by calcineurin. Proc Natl Acad Sci U S A. 1995;92(7):2682–2685. doi:10.1073/pnas.92.7.2682.

[150] Herman SB, Juilfs DM, Fauman EB, Juneau P, Menetski JP. Analysis of a mutation in phosphodiesterase type 4 that alters both inhibitor activity and nucleotide selectivity. Mol Pharmacol. 2000;57(5):991–999.

[151] Sharma RK, Wang JH. Regulation of cAMP concentration by calmodulin-dependent cyclic nucleotide phosphodiesterase. Biochem Cell Biol. 1986;11(64):1072–80.

[152] Sharma RK, Kalra J. Molecular interaction between cAMP and calcium in calmodulin-dependent cyclic nucleotide phosphodiesterase system. Clin Invest Med. 1994;4(17):374–382.
