# Supplementary material for: β-adrenergic signaling broadly contributes to LTP induction
Source: PLoS Comput Biol. 2017 Jul 24;13(7):e1005657. doi: 10.1371/journal.pcbi.1005657 (PMC5546712; doi:10.1371/journal.pcbi.1005657)
Supplement: S2 Table — Abbreviations same as in S1 Table. (PDF) [file pcbi.1005657.s002.pdf]

Table S2: **Diffusion rates of diffusible species. Abbreviations same as in S1 Table.**

Molecular species	k_{diff} $\left[\frac{\mu m^2}{s}\right]$
Ca	174.20
Ca_{Out}	2.00
Calbindin	9.25
Calbindin-Ca	9.25
CaBuffer	10.00
CaBuffer-Ca	10.00
NEBuffer	10.00
NE	111.40
Epac	5.21
EpaccAMP	5.20
ATP	1
cAMP	86.4
AMP	84.8
CaM	11
$CaMCa_2$	11.00
$CaMCa_4$	11.00
CaMKII	7.62
$CaMKIICaMCa_4$	0.10
$pCaMKIICaMCa_4$	0.10
pCaMKII	0.10
PKAc	0.836
Inhibitor-1	9.49
Inhibitor-1PKAc	10.6
Ip35	7.02
Carvedilol	80.50
ICI-118,551	91.40
Propranolol	95.84
