# Supplementary material for: β-adrenergic signaling broadly contributes to LTP induction
Source: PLoS Comput Biol. 2017 Jul 24;13(7):e1005657. doi: 10.1371/journal.pcbi.1005657 (PMC5546712; doi:10.1371/journal.pcbi.1005657)
Supplement: S3 Table — Initial conditions of remaining cytosolic species (Complex, pComplex, I1PKAc, PKAc-PDE4-cAMP, CaBCa, PDE1CaMCa4cAMP, Ip35PP2BCaMCa4, L, pPDE4-cAMP, PKAc, CaB) were set to 0. (PDF) [file pcbi.1005657.s003.pdf]

Table S3: **Initial conditions of cytosolic species.** Initial conditions of remaining cytosolic species (Complex, pComplex, I1PKAc, PKAc-PDE4-cAMP, CaBCa, $PDE1CaMCa_4cAMP$, $Ip35PP2BCaMCa_4$, L, pPDE4-cAMP, PKAc, CaB) were set to 0.

Molecule name	general cytosol [nM]
L	9.0
LOut	2500014.0
Ca	71.0
CaOut	1875021.0
Calbin	145713.0
CalbinC	14641.0
ATP	1992779.0
AMP	980.0
cAMP	58.0
Epac1	488.0
Epac1cAMP	12.0
PDE1	11468.0
$PDE1CaMCa_4$	295.0
CaM	10959.0
$CaMCa_2$	120.0
$CaMCa_4$	3.0
NgCaM	17992.0
Ng	2139.0
PP2BCaM	2573.0
PP2BCaMCa2	329.0
$PP2BCaMCa_4$	3.0
CK	21269.0
$CKCaMCa_4$	9.0
$CKpCaMCa_4$	1061.0
$CKpCaMCa_4PP1$	9.0
CKp	870.0
CKpPP1	9.0
I1	732.0
Ip35	9.0
PP1	1122.0
Ip35PP1	443.0
$PP1PP2BCaMCa_4$	12.0
