# Supplementary material for: β-adrenergic signaling broadly contributes to LTP induction
Source: PLoS Comput Biol. 2017 Jul 24;13(7):e1005657. doi: 10.1371/journal.pcbi.1005657 (PMC5546712; doi:10.1371/journal.pcbi.1005657)
Supplement: S4 Table — Initial conditions of remaining anchored species (LRGs, LRGsβγ, pR, pLR, ppLR, pppLR, ppppLR, pR, ppR, pppR, ppppR, PKAcR, PKAcpR, PKAcppR, PKAcpppR, PKAcLR, PKAcpLR, PKAcppLR, PKAcpppLR, ppppLRGi, ppppRGi, ppppRGiβγ, ppppLRGiβγ, Giβγ, Gαi GDP, AC1Gαs GTPGαi GTPCaMCa4, AC1Gαs GTPGαi GTP, Gαi GTP AC1Gαi GTPCaMCa4, AC1GsGiCaMCa4ATP, AC1Gαs GTPCaMCa4, AC1Gαi GTP, AC1Gαs GTPCaMCa4 ATP AC1Gαi GTPCaMCa4 ATP,) were set to 0 both in the spinehead and in the dedritic submembrane. (PDF) [file pcbi.1005657.s004.pdf]

Table S4: **Initial conditions of species anchored in the spine head and in the dendrite membrane.** Initial conditions of remaining anchored species (LRG_s,$LRG_{s\beta\gamma}$, pR, pLR, ppLR, pppLR, ppppLR, pR, ppR, pppR, ppppR, PKAcR, PKAcpR, PKAcppR, PKAcpppR, PKAcLR, PKAcpLR, PKAcppLR, PKAcpppLR, $ppppLRG_i$, $ppppRG_i$, $ppppRG_{i\beta\gamma}$, $ppppLRG_{i\beta\gamma}$, $G_{i\beta\gamma}$,$G_{\alpha i}GDP$, $AC1G_{\alpha s}GTPG_{\alpha i}GTPCaMCa_4$, $AC1G_{\alpha s}GTPG_{\alpha i}GTP$, $G_{\alpha i}GTP$ $AC1G_{\alpha i}GTPCaMCa_4$, $AC1GsGiCaMCa_4ATP$, $AC1G_{\alpha s}GTPCaMCa_4$, $AC1G_{\alpha i}GTP$, $AC1G_{\alpha s}GTPCaMCa_4ATP$ $AC1G_{\alpha i}GTPCaMCa_4ATP$,) were set to 0 both in the spinehead and in the dedritic submembrane.

Anchored molecules	spine cytosol [nM]	dendrite focal membrane [nM]
R	42	27
G_sR	1134	758
G_s	2856	1926
G_i	2000	1322
AC1	18150	2724
AC1CaMCa4	63	2
AC1CaMCa4ATP	900	41
AC8	49170	751
PKA	4000	357
PKAcAMP2	1848	5
PKAcAMP4	100	0
PDE4	450	750
pPDE4	600	300
