# Supplementary material for: β-adrenergic signaling broadly contributes to LTP induction
Source: PLoS Comput Biol. 2017 Jul 24;13(7):e1005657. doi: 10.1371/journal.pcbi.1005657 (PMC5546712; doi:10.1371/journal.pcbi.1005657)
Supplement: S5 Table — Initial conditions of remaining anchored species (Leak, ncx, ncxCa, pmca, pmcaCa, GluR1-CKCam, GluR1-CKp, GluR1-CKpCaM, GluR1-PKAc, pS831GluR1, pS831-GluR1-PKAc, pS831GluR1-PP1, pS845GluR1, pS845GluR1-CKCam, pS845GluR1-CKp, pS845GluR1-CKpCaM, pS845GluR1-PP1, pS845GluR1-PP2B, pS845pS831GluR1, pS845pS831GluR1-PP1, pS845pS831GluR1-PP2B). (PDF) [file pcbi.1005657.s005.pdf]

Table S5: **Initial conditions of species anchored in the PSD.** Initial conditions of remaining anchored species (Leak, ncx, ncxCa, pmca, pmcaCa, GluR1-CKCam, GluR1-CKp, GluR1-CKpCaM, GluR1-PKAc, pS831GluR1, pS831-GluR1-PKAc, pS831GluR1-PP1, pS845GluR1, pS845GluR1-CKCam, pS845GluR1-CKp, pS845GluR1-CKpCaM, pS845GluR1-PP1, pS845GluR1-PP2B, pS845pS831GluR1, pS845pS831GluR1-PP1, pS845pS831GluR1-PP2B).

Anchored molecules	PSD [nM]
CaOutLeak	700.0
GluR1	13100.0
PDE4	450.0
pPDE4	600.0
