# Supplementary material for: β-adrenergic signaling broadly contributes to LTP induction
Source: PLoS Comput Biol. 2017 Jul 24;13(7):e1005657. doi: 10.1371/journal.pcbi.1005657 (PMC5546712; doi:10.1371/journal.pcbi.1005657)
Supplement: S6 Table — (PDF) [file pcbi.1005657.s006.pdf]

Table S6: **Parameters of the norepinephrine release model fitted to (1).**

parameter	value
U_{SE}	0.003
τ_{f}	0.48 s
τ_{r}	0.31 s
τ_{i}	3.58 s
A_{SE}	328 nM
