# Supplementary material for: β-adrenergic signaling broadly contributes to LTP induction
Source: PLoS Comput Biol. 2017 Jul 24;13(7):e1005657. doi: 10.1371/journal.pcbi.1005657 (PMC5546712; doi:10.1371/journal.pcbi.1005657)
Supplement: S7 Table — The kinase-to-phosphatase balance, evaluated by molecular signatures, is thought to control direction of synaptic plasticity. There are at least two ways of assessing this balance: either measuring the quantity of phosphorylated targets of kinases and phosphatases, or assessing a ratio of kinase activity to phosphatase activity. In this table spine molecular signature has an alternative form and evaluates Epac activity and the ratio of active CaMKII and active PKA to active phosphatases (PP1 and PP2B). This form of spine signature is very noisy, hence to induce spine specific changes, the spine signature has to exceed its threshold for 10 sec uninterrupted. (PDF) [file pcbi.1005657.s007.pdf]

Table S7: **Robustness of the spine signature.** The kinase-to-phosphatase balance, evaluated by molecular signatures, is thought to control direction of synaptic plasticity. There are at least two ways of assessing this balance: either measuring the quantity of phosphorylated targets of kinases and phosphatases, or assessing a ratio of kinase activity to phosphatase activity. In this table spine molecular signature has an alternative form and evaluates Epac activity and the ratio of active CaMKII and active PKA to active phosphatases (PP1 and PP2B). This form of spine signature is very noisy, hence to induce spine specific changes, the spine signature has to exceed its threshold for 10 sec uninterrupted.

stimulation paradigm	uniterrupted above the lower threshold	uniterrupted above the higher threshold
LFS	0/4	0/4
ISO	1/4	0/4
HFS	4/4	4/4
4xHFS-3s	8/8	8/8
4xHFS-80s	8/8	8/8
ISO+HFS	4/4	4/4
ISO+LFS	4/4	4/4
HFS no PKA	1/4	0/4
ISO+HFS no PKA	8/8	8/8
4xHFS-80s no PKA	8/8	8/8
4xHFS-3s no PKA	4/4	4/4
ISO+LFS no PKA	1/4	0/4
Propranolol+4xHFS	8/8	8/8
ICI-118551+4xHFS	4/4	4/4
Carvedilol+HFS	4/4	4/4
Carvedilol+LFS	0/4	0/4
Carvedilol+2xHFS	4/4	4/4
Carvedilol+3xHFS	4/4	4/4
