# Supplementary material for: β-adrenergic signaling broadly contributes to LTP induction
Source: PLoS Comput Biol. 2017 Jul 24;13(7):e1005657. doi: 10.1371/journal.pcbi.1005657 (PMC5546712; doi:10.1371/journal.pcbi.1005657)
Supplement: S8 Table — (PDF) [file pcbi.1005657.s008.pdf]

Table S8: Robustness of the spine signature threshold. Comparison of number of spine signatures above the threshold for 10 sec and 15 sec.

stimulation paradigm	10 sec above the lower threshold	15 sec above the lower threshold	10 sec above the higher threshold	15 sec above the higher threshold
LFS	0/4	0/4	0/4	0/4
ISO	0/4	0/4	0/4	0/4
HFS	4/4	4/4	4/4	4/4
4xHFS-3s	8/8	8/8	8/8	8/8
4xHFS-80s	8/8	8/8	8/8	8/8
ISO+HFS	4/4	4/4	4/4	4/4
ISO+LFS	4/4	4/4	4/4	4/4
HFS no PKA	0/4	0/4	0/4	0/4
ISO+HFS no PKA	8/8	8/8	8/8	7/8
4xHFS-80s no PKA	8/8	8/8	8/8	8/8
4xHFS-3s no PKA	4/4	4/4	4/4	4/4
ISO+LFS no PKA	0/4	0/4	0/4	0/4
Propranolol+4xHFS	8/8	8/8	8/8	8/8
ICI-118551+4xHFS	4/4	4/4	4/4	4/4
Carvedilol+HFS	4/4	2/4	0/4	0/4
Carvedilol+LFS	0/4	0/4	0/4	0/4
Carvedilol+2xHFS	4/4	4/4	4/4	4/4
Carvedilol+3xHFS	4/4	4/4	4/4	4/4
